# Coordinated Multicellular Immune Programs and Drug Targets Revealed by Single-Cell Analysis in Driver-Mutated NSCLC

**DOI:** 10.3390/ijms27093997

**Published:** 2026-04-29

**Authors:** Kuan Yang, Kaiyue Yang, Jiasi Wang, Hang Zhao, Wenqi Jiang, Depeng Mu, Xiao Peng, Yiming Yan, Xing Gao, Jing Bai, Congxue Hu, Yunpeng Zhang, Xia Li

**Affiliations:** 1 College of Bioinformatics Science and Technology, Harbin Medical University, Harbin 150081, China; yangkuan@hrbmu.edu.cn (K.Y.); yangkaiyue0904@hrbmu.edu.cn (K.Y.); 2023020627@hrbmu.edu.cn (J.W.); 2024020599@hrbmu.edu.cn (H.Z.); 202301206@hrbmu.edu.cn (W.J.); 2023020607@hrbmu.edu.cn (D.M.); 2025020433@hrbmu.edu.cn (X.P.); 2025020493@hrbmu.edu.cn (Y.Y.); gx011018@hrbmu.edu.cn (X.G.); baijing@hrbmu.edu.cn (J.B.); hucx1996@hrbmu.edu.cn (C.H.); 2 School of Intelligent Medicine and Technology, Big Data Research Center, Hainan Medical University, No. 3 Xueyuan Road, Longhua District, Haikou 571199, China

**Keywords:** driver mutated, immune coordination, malignant regulatory network, prognostic biomarkers, single-cell transcriptomics

## Abstract

Oncogenic driver mutations in non-small cell lung cancer (NSCLC) activate defined signaling pathways that sustain tumor growth and influence the immune landscape. Yet, how coordinated interactions among diverse cell populations within the tumor immune microenvironment (TIME) contribute to this process remains largely unresolved. To address this, we profiled approximately 200,000 single cells from 45 treatment-naïve NSCLC patients representing seven major driver mutations. This analysis uncovered five multicellular modules (CM1–5) with distinct functional properties, each linked to specific malignant regulatory programs. Among them, CM2 and CM5 exhibited pronounced invasive features and were associated with unfavorable clinical outcomes. CM2 was predominantly observed in *EGFR*- and *MET*-driven brain metastases and was defined by strong crosstalk between astrocytes and myofibroblasts. Factors such as SPP1, PTN, and PSAP, together with metabolic alterations, contributed to a microenvironment supportive of metastatic colonization in the brain. By contrast, CM5 was enriched in *ROS1*-, *KRAS*-, and *EGFR*-mutant tumors and consisted of diverse myeloid and endothelial subsets characterized by immunosuppressive and pro-angiogenic signaling, including MIF, GALECTIN, and RETN, collectively facilitating immune escape and vascular remodeling. We further constructed and validated a driver mutation-specific prognostic signature (DMSP.sig) model integrating receptor–ligand interactions and core transcription factors, which effectively stratified patient survival. Leveraging this model, we also identified potential therapeutic candidates linked to these prognostic features, highlighting opportunities for clinical intervention. In summary, our study delineates how oncogenic drivers give rise to distinct TIME architectures, providing a framework for prognostic assessment and precision immunotherapy in high-risk NSCLC.

## 1. Introduction

Non-small cell lung cancer (NSCLC) is the most prevalent histological subtype of lung cancer, accounting for approximately 85% of all cases [1]. Its pronounced biological heterogeneity is manifested across multiple dimensions, including genetic alterations, cellular origins, tumor microenvironment, and immune responses [2]. Advances in high-throughput sequencing technologies, particularly next-generation sequencing (NGS), have enabled comprehensive molecular characterization of NSCLC, leading to the identification of numerous oncogenic alterations, several of which have become critical targets for therapeutic development [3]. These discoveries have substantially advanced the field of precision oncology in NSCLC, fostering a novel classification paradigm that emphasizes molecularly defined subtypes to guide the rational implementation of targeted therapies [4].

Multiple oncogenic driver mutations have been identified in NSCLC patients, including alterations in epidermal growth factor receptor (*EGFR*), anaplastic lymphoma kinase (*ALK*), and the receptor tyrosine kinase *ROS1*. Therapeutic agents such as monoclonal antibodies and small-molecule inhibitors targeting key signaling pathways that regulate tumor growth and progression have shown promise in improving patient survival and quality of life. Notably, the distribution of driver mutations exhibits histological specificity: mutations in *EGFR*, *KRAS*, *BRAF*, *ALK*, and *ROS1* are predominantly observed in lung adenocarcinoma, whereas alterations in *FGFR1*, *DDR2*, and components of the PI3K pathway are more commonly detected in squamous cell carcinoma. Furthermore, NGS-based studies have revealed that individual tumors can harbor concurrent double, triple, or even multiple co-occurring mutations, which may interact to influence therapeutic response and drug sensitivity [5]. Thus, a deeper understanding of the molecular heterogeneity of NSCLC is essential for uncovering novel pathogenic mechanisms and optimizing individualized treatment strategies.

Certain oncogenic events, such as mutations in *KRAS*, *TP53*, and *MET*, have been demonstrated to possess prognostic relevance and potential predictive value for neoadjuvant therapy [6,7,8]. In addition, differences in driver mutations can profoundly reshape the composition of the tumor immune microenvironment (TIME). Preclinical studies have shown that NSCLC driven by *EGFR* or *KRAS* mutations displays distinct immune cell infiltration patterns in terms of both cellular composition and phenotypic characteristics [9,10].

In recent years, the advent of single-cell RNA sequencing (scRNA-seq) has enabled high-resolution dissection of cellular heterogeneity in NSCLC. This technology has facilitated the construction of comprehensive cellular atlases within NSCLC tumor tissues and has highlighted the critical role of TIME in mediating responses to therapeutic interventions [11]. Specifically, immune components within TIME—including exhausted CD8^+^ T cells, M2-like tumor-associated macrophages (TAMs), and regulatory B cells—play key roles in tumor progression and responsiveness to immunotherapy, and have emerged as promising biomarkers for predicting immunotherapeutic outcomes [12,13].

In this study, we integrated single-cell RNA sequencing data from 45 treatment-naïve patients with driver-mutated NSCLC, classified into 10 subtypes based on primary versus metastatic status and driver mutations. Using these data, we constructed a TIME atlas and identified functionally specialized immune coordination programs and driver mutation specific prognostic signature (DMSP.sig) to facilitate patient risk stratification, immune profiling, and prognostic prediction. These findings provide new insights into the molecular and immune mechanisms of NSCLC and offer a framework for precision therapy development.

## 2. Results

### 2.1. A Single-Cell Atlas in Non-Small Cell Lung Cancer (NSCLC) Harboring Driver Mutations

**Figure 1:**
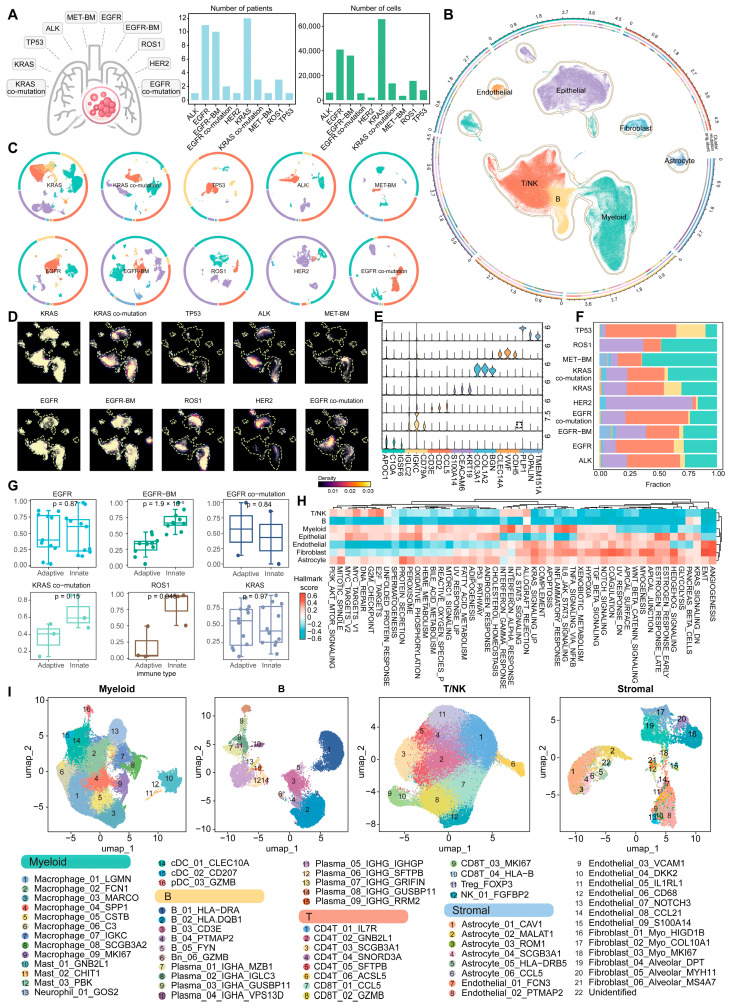
A comprehensive single-cell atlas of driver-mutated Non-small cell lung cancer (NSCLC). (**A**) Data overview. Bar charts show the number of patients and the number of cells for each driver mutation subtype of NSCLC. (**B**) Uniform Manifold Approximation and Projection unveiling seven major cell lineages, and colors represent different cell populations. The concentric ring plot displays, from the innermost to the outermost layer, cell types, oncogenic driver mutations, and individual patients. (**C**) UMAP plots of 10 driver-mutated NSCLC samples, colors represent different cell populations. The outer ring indicates the proportion of each cell type. (**D**) UMAP density plots of 10 driver-mutated NSCLC samples, colors represent the intensity of cell density. (**E**) Violin plots showing the expression of marker genes across seven major cell types. (**F**) Stacked bar plot showing the distribution of major cell types in each driver-mutated NSCLC. (**G**) Boxplots of innate (myeloid) and adaptive (T/B) immune cell proportions across driver mutations. Subgroups with a sample size n > 1 are presented: *EGFR* (n = 11), *EGFR*-BM (n = 10), *EGFR* co-mutation (n = 2), *KRAS* co-mutation (n = 3), *ROS1* (n = 3) and *KRAS* (n = 12). Statistical significance was determined using the Kruskal–Wallis test. (**H**) GSVA heatmap of hallmark pathway enrichment across seven cell types. (**I**) UMAP plots of tumor immune microenvironment (TIME) clusters from 10 driver-mutated NSCLC samples, displayed in panels according to myeloid cells, B cells, T/NK cells, and stromal cells.

To comprehensively dissect the heterogeneity of TIME in NSCLC harboring distinct oncogenic driver mutations, we systematically integrated single-cell RNA sequencing (scRNA-seq) data from 45 treatment-naive NSCLC patients (Methods). These tumors harbored specific oncogenic driver mutations, including *EGFR*, *KRAS*, *ROS1*, *ALK*, and others (Figure 1A; Table S1). Among them, *EGFR* and *KRAS* mutations were the most common and were often accompanied by co-mutations with other genes. Given the potential of these driver mutations to promote brain metastasis [14], we further incorporated scRNA-seq data from NSCLC patients with brain metastases into our study (Figure S1B).

Following rigorous quality control and Harmony-based integration, we obtained high-quality transcriptional profiles for 196,560 cells, which were classified into seven major lineages using canonical marker genes: epithelial cells, endothelial cells, fibroblasts, astrocytes, T/NK cells, B cells, and myeloid cells (Figure 1B,E and Figure S1A, Methods). Concurrently, we observed that cells from different datasets and driver subtypes consistently clustered by cell type, with each cluster comprising cells from multiple patients, indicating the conservation of major cell types across NSCLC with distinct driver mutations and the robustness of data integration (Figure S1B).

Through in-depth dissection of cellular composition patterns in NSCLC harboring distinct driver mutations, we found that immune cells comprise a significant proportion across all driver subtypes (Figure 1C,D,F and Figure S1C), while exhibiting distinct compositional patterns that reveal heterogeneous immunological landscapes across different mutational contexts. Notably, NSCLC with *EGFR* brain metastases (*EGFR*-BM) and *ROS1*-mutant exhibited significantly lower lymphocyte proportions compared with myeloid cell infiltration (Figure 1G), clearly indicating dominance of innate immunity in their tumor immune microenvironment (TIME) [15] (Methods). In addition, we observed that endothelial cells, fibroblasts, and astrocytes were markedly enriched in malignant signaling pathways, such as angiogenesis and epithelial–mesenchymal transition (EMT) [16], whereas T/NK cells and myeloid cells predominantly exhibited enrichment in interferon-α and interferon-γ pathways [17]. These findings suggest that stromal and immune cells within the TIME may promote cancer progression and invasion through distinct oncogenic signaling axes (Figure 1H).

To further elucidate the intrinsic heterogeneity and degree of malignancy of the TIME, we performed reclustering of its major components, including immune cells (myeloid, B, and T/NK cells) and stromal cells (astrocytes, endothelial cells, and fibroblasts), and generated a high-resolution atlas comprising 64 cellular subsets that comprehensively delineates the cellular composition of the TIME (Figure 1I; Table S2). Notably, these TIME cell subsets exhibited driver mutation-specific distribution patterns in NSCLC (Figure S1D, Method). For example, astrocyte subsets were predominantly enriched in *EGFR*-BM patients, whereas distinct B cell and plasma cell subsets were more prominently enriched in patients harboring *KRAS* and *TP53* mutations, respectively. In particular, B cells expressing *HLA-DRA* and *HLA-DQB1*, which are closely associated with MHC class II antigen presentation [18], suggest the presence of a potential B cell–mediated antitumor immune response. Collectively, we established a comprehensive single-cell atlas of NSCLC with driver mutations, revealing TIME cell subsets with subtype-specific distribution patterns.

### 2.2. Cellular Module Analyses Reveal Five Tumor Immune Microenvironment (TIME) Subtypes

**Figure 2:**
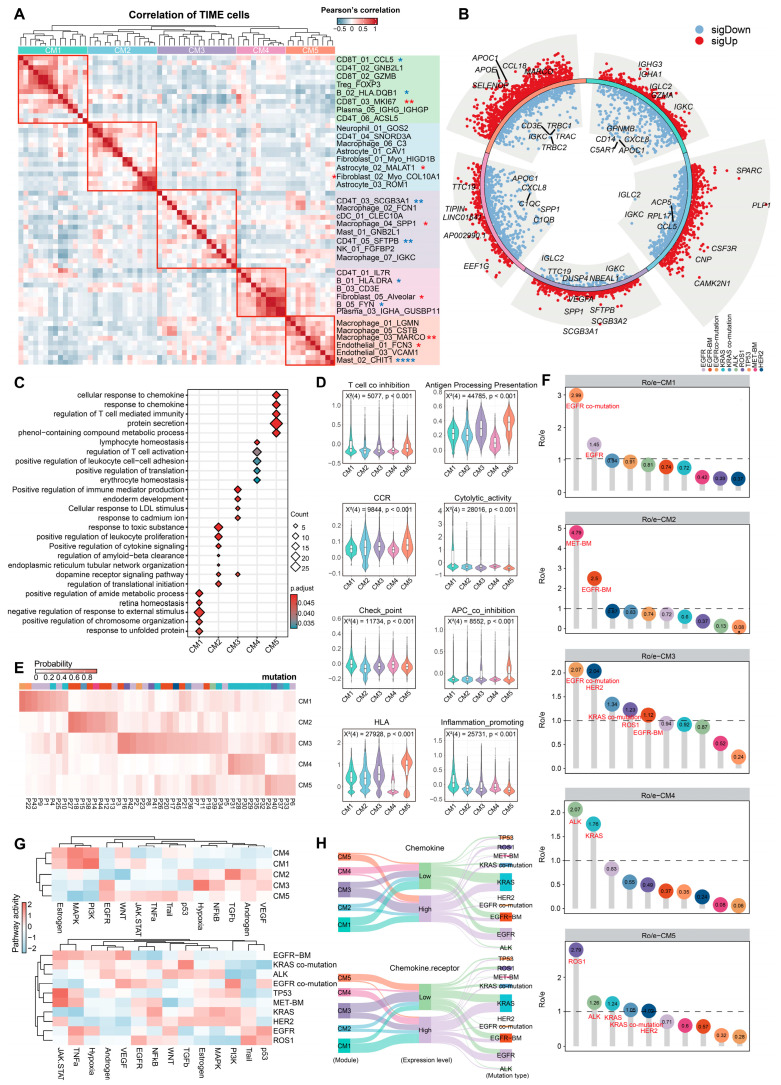
Five distinct tumor immune microenvironment (TIME) subtypes identified in driver-mutated NSCLC. (**A**) The five cell modules derived from correlation analysis among major cell clusters within the TIME, with key contributing cell clusters for each module shown on the right. The number of asterisks indicates the level of statistical significance, with blue representing favorable prognosis and red representing unfavorable prognosis. The significance levels are defined as * *p* < 0.05, ** *p* < 0.01, and **** *p* < 0.0001. (**B**) Volcano plots showing differentially expressed genes (DEGs) across the five TIME subtypes, with the top five marker genes labeled. Wilcoxon rank-sum test, adjusted *p* value < 0.05. (**C**) Dot heatmap showing enriched pathways across TIME subtypes. Benjamini–Hochberg-adjusted hypergeometric test. (**D**) Violin plots showing the scores of various immune gene sets across TIME subtypes. Chi-square test for statistical comparison. (**E**) Heatmap showing the proportions of each TIME subtype across NSCLC patients. (**F**) Lollipop plot showing the tissue distribution preference of each TIME subtype across different driver-mutated NSCLC samples. Dot colors represent the type of driver-mutated NSCLC samples, and the values represent the Ro/e ratio. (**G**) Heatmap showing the enrichment levels of 14 oncogenic pathways in TIME subtypes (top) and driver-mutated NSCLC samples (bottom). (**H**) Sankey plots illustrating the associations of chemokine (top) and chemokine receptor (bottom) expression patterns with TIME subtypes and driver-mutated NSCLC sample types. Expression levels are categorized as high or low based on the median value.

Given the pivotal role of the heterogeneous composition of immune and stromal cells in shaping the tumor immune landscape [19], we conducted a co-occurrence analysis of TIME cells in NSCLC patients with distinct driver mutations and identified five robust multicellular modules (CM1–5) exhibiting co-occurrence patterns (Figure 2A; Table S3; Methods). Strikingly, diverse immune and stromal cell subsets associated with prognostic risk coexisted within the same module, suggesting that each module represents a distinct TIME subtype with potential impact on tumor progression (Table S4).

To further delineate the characteristics of each TIME subtype in tumor immunity, we performed a systematic analysis by integrating differentially expressed genes, functional enrichment results, and immune-related gene set scores (Figure 2B–D and Figure S2A,B, Table S5, Methods). CM1, characterized by high lymphocyte infiltration, exhibited pronounced upregulation of immunoglobulin-related genes, including *IGHA1*, *IGHG3*, and *IGKC*, potentially linked to antitumor immunity [20]. Moreover, enrichment of the unfolded protein response (UPR) pathway in CM1 suggests potential alterations in T cell phenotype and function [21], and its significantly elevated scores in T cell differentiation, antigen receptor-mediated signaling, and cytotoxic/proinflammatory signaling collectively indicate that CM1 represents a lymphocyte-mediated, immune-activated TIME. In contrast, CM2 exhibited a complex cellular composition, including co-enriched multiple astrocyte subtypes, myofibroblasts, and heterogeneous immune cell subsets. Upregulation of *SPARC* may be linked to APA-mediated mechanisms of cancer progression in tumor-associated fibroblasts [22], whereas elevated *PLP1* suggests the presence of Schwann cell-mediated angiogenesis promoting tumor progression within the TIME [23]. Additionally, enrichment of cytokine-positive regulation and elevated expression of CC chemokines may act as driving forces of malignant progression. The enrichment of glial cell development, differentiation, and generation further supports astrocyte presence, collectively characterizing CM2 as a malignant TIME driven by astrocyte-myofibroblast interactions.

Interestingly, CM3 exhibited high myeloid and T cell infiltration, whereas the immunosuppressive Macrophage_04_SPP1 subset may promote metastasis via angiogenesis and ECM remodeling [24]. CM3 also exhibited specific expression of the tumor suppressors *SCGB3A1* and *SCGB3A2* [25], as well as *SFTPB*, a gene associated with lung cancer risk [26], alongside NK_01_FGFBP2 enrichment linked to major pathological response [27]. Functionally, CM3 was enriched in MHC class II-mediated antigen presentation and positive regulation of leukocyte proliferation. Collectively, CM3 harbors both immune-activating signals and immunosuppressive elements, indicating a dynamic state where antitumor immunity and immune evasion coexist. CM4 characterized high infiltration of lymphocytes and alveolar fibroblasts, with marked upregulation of *TTC19*, involved in cell division [28], and *EEF1G*, linked to translation [29], alongside enrichment in ribosome biogenesis, translation-related pathways, and signals regulating lymphocyte homeostasis and T cell activation. However, its relatively low antigen presentation score suggests impaired antigen presentation or reliance on non-classical pathways for T cell activation, indicating an “activated but insufficient” immune state. In contrast, CM5 was dominated by diverse myeloid and endothelial cell subsets, with notable upregulation of macrophage-related marker genes and chemokine signaling, and enrichment in MHC class II-mediated antigen presentation and pathways regulating T cell-mediated immunity. Nonetheless, its elevated antigen-presenting cell (APC) co-inhibition score points to mechanisms that suppress T cell-mediated antitumor responses [30]. Therefore, CM5 represents an immunosuppressive TIME subtype that exhibits enhanced antigen presentation while concomitantly restraining T cell functionality.

To further investigate the association between the five TIME subtypes with distinct immune states and driver-mutant NSCLC, we assessed the most predominant TIME subtype in each patient (Figure 2E; Methods). The immune high-infiltration subtypes CM1 and CM3 were enriched in *EGFR*-driven patients, whereas CM2 was preferentially enriched in those with *EGFR*-BM, suggesting that brain metastasis is accompanied by remodeling of the immune microenvironment, consistent with previous findings [31]. Moreover, CM3 was not only the most prevalent TIME subtype across the cohort but may also represent a more general immune state; CM4 was predominantly enriched in *KRAS*-driven tumors, suggesting it may be dominated by a specific oncogenic pathway; while CM5 encompasses patients with diverse driver alterations such as *ROS1*, *KRAS*, and others, hinting that it represents an immune subtype characterized by higher heterogeneity and more complex underlying mechanisms. Further analysis of tissue preference also confirmed these distribution patterns (Figure 2F).

Owing to oncogenic signaling pathways, cytokines, and chemokines can profoundly influence the cellular composition and immune phenotype of the TIME [32]. To further investigate the mechanisms underlying the heterogeneous preference patterns of different TIME subtypes, we evaluated malignant pathway activity in each TIME subtype and driver-mutant NSCLC (Figure 2G) and analyzed the expression patterns of chemokines (Figure 2H). Distinct malignant pathway enrichment patterns were observed across TIME subtypes and driver-mutant NSCLC. In particular, CM1 and CM4 exhibited highly similar malignant pathway enrichment features, including key pathways such as *PI3K* and *MAPK*, accompanied by a pattern of upregulated chemokine expression and downregulated receptor expression. These findings suggest that chemokine expression patterns may play a critical role in shaping malignant pathways, thereby influencing the subtype-specific distribution of the TIME in driver-mutant NSCLC [33].

In summary, we identified five TIME subtypes with distinct immune states in driver-mutant NSCLC, characterized by multicellular co-occurrence, functional heterogeneity, and specifically malignant pathway enrichment, collectively reflecting patterns of immune microenvironment remodeling and regulatory mechanisms in the context of oncogenic drivers.

### 2.3. Cellular Module (CM)2 and CM5 Are Associated with Poor Prognosis

**Figure 3:**
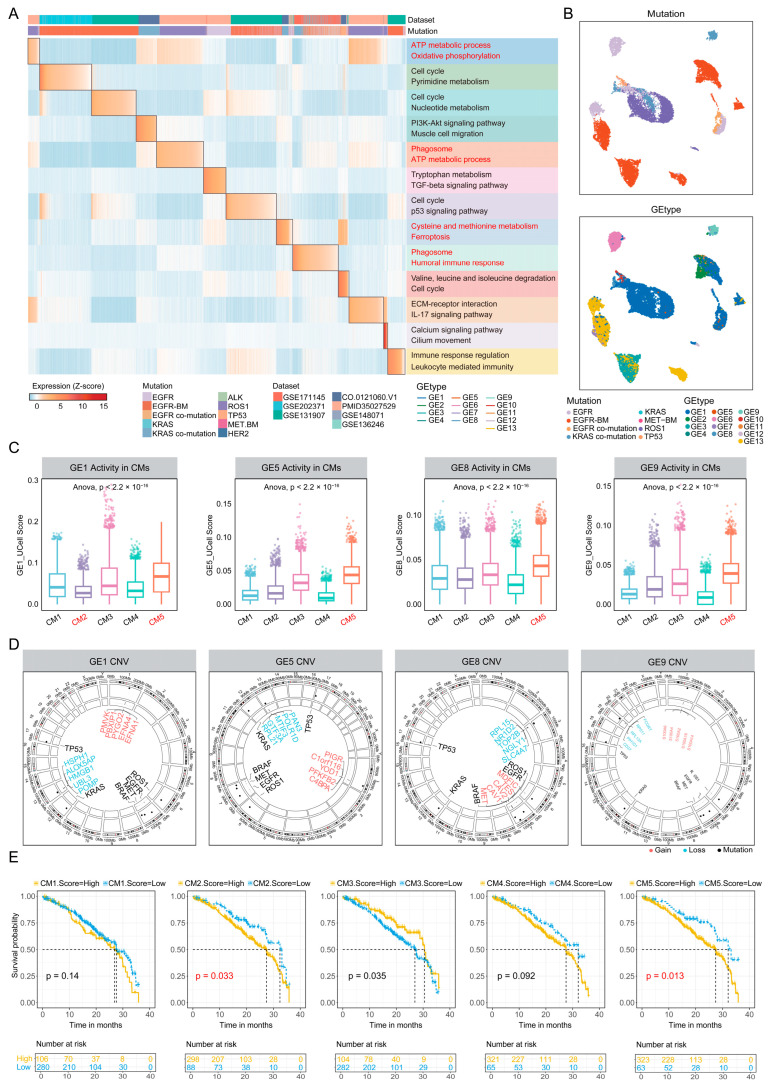
Characterization of malignant features of cancer epithelial cells across TIME subtypes. (**A**) Heatmap of Z-scored signature scores of the 13 identified gene elements (GEs) across all cancer epithelial cells. Annotations indicate dataset origin and driver-mutated NSCLC subtype. The right panel shows the main functions enriched in each GE. (**B**) UMAP visualization of cancer epithelial cells, colored by driver-mutated NSCLC subtype (top) and by GE-defined classification (bottom). (**C**) Boxplots showing the distribution of representative GEs signature scores across TIME subtypes. Statistical significance was assessed using ANOVA. (**D**) Circo plot of the genome showing major CNV events within representative GEs. Top 5 amplified genes are shown in red, top 5 deleted genes in blue, and known driver genes in black. (**E**) Progression-free survival (PFS) over three years stratified by TIME subtype. Patients were categorized into high-score and low-score groups based on optimal threshold functions for each subtype (CM1 high: n = 106, low: n = 280; CM2 high: n = 298, low: n = 88; CM3 high: n = 104, low: n = 282; CM4 high: n = 321, low: n = 65; CM5 high: n = 323, low: n = 63). Statistical significance was determined using log-rank tests.

Due to cancer cells interacting with the TIME through multiple oncogenic pathways, they form complex signaling networks that influence immune infiltration and patient outcomes [34,35]. To elucidate the intrinsic determinants underlying the heterogeneous distribution of different TIME subtypes, we first performed a systematic analysis focusing on the central origin of oncogenic pathways—cancer cells. Based on tumor cells identified by Copycat in the integrated dataset, we conducted multiresolution unsupervised clustering by sample and extracted upregulated marker genes for each cluster to delineate their principal molecular features. Through consensus clustering, we identified 13 gene elements (GEs) representing the key molecular characteristics of NSCLC cells with distinct driver mutations (Figure S3C,D; Table S6; Methods). Using these GEs, each cancer cell was scored and assigned a predominant cell state (Figure 3A).

Notably, GE1 exhibited elevated ATP metabolism and oxidative phosphorylation activities, features that may be associated with a favorable prognosis [36] (Table S7). In contrast, subgroups such [34,35] as GE2, GE3, and GE7 displayed pronounced cell-cycle activity and enhanced metabolic states, suggesting that they are in a highly proliferative phase and potentially possess stronger division and invasive capabilities. GE9 was characterized by high levels of phagosome activity and humoral immune responses, implying that it may reside within a microenvironment enriched with tertiary lymphoid structures (TLSs), accompanied by substantial B cell infiltration and potentially linked to augmented anti-tumor immune responses [37]. Further dimensionality reduction and clustering analyses of cancer cells revealed pronounced intra- and inter-tumoral heterogeneity in cancer cell states across NSCLC harboring distinct driver mutations (Figure 3B). In *EGFR*-driven tumors, the predominant states included GE1 with high oxidative phosphorylation, GE6 with TGF-β–associated programs, and GE13 enriched for leukocyte-mediated immune responses, indicating the coexistence of pro-invasive and antitumor immune activities. Consistent with previous studies, *EGFR* signaling suppressed CD8^+^ T cell infiltration and conferred resistance to *PD-1* blockade via ERK1/2–P90RSK–TGF-β activation [38], thereby fostering an immunosuppressive TIME. In *EGFR* brain metastases, beyond the GE1 and GE13 states observed in primary tumors, two additional states emerged: GE2, characterized by high proliferative and pyrimidine metabolism activity consistent with medulloblastoma [39], and GE3, characterized by nucleotide metabolism-driven high-proliferation and invasive behaviors observed across multiple cancer types [40]. These findings suggest substantial remodeling of cancer cell states during brain metastasis in *EGFR*-driven NSCLC. In *KRAS*-mutant patients, the cancer cells were relatively rare. However, in those with *KRAS* co-mutations, in addition to GE1 with high oxidative phosphorylation, GE8 with cysteine metabolism-mediated ferroptosis resistance [41], and GE9 with pronounced humoral immune activity also emerged. These findings indicate that in *KRAS* co-mutant patients, cancer cells may employ a dual adaptive strategy, involving metabolic reprogramming to resist oxidative stress and ferroptosis, while maintaining an immune-active state [42]. Distinct from the above subtypes, *ROS1*-mutant NSCLC was dominated by the GE1 subgroup with ATP metabolism and oxidative phosphorylation, showing a relatively homogeneous state with limited functional diversity.

To further investigate the potential influence of distinct GEs on TIME phenotypes, we conducted gene set scoring of 13 GE states in TIME cells. The results revealed that GE1, GE5, GE8, and GE9 exhibited significant associations with specific TIME subtypes (Figure 3C), suggesting crucial roles in shaping the TIME. Strikingly, CM2 showed a relative paucity of GE1 cancer cells with low oxidative phosphorylation activity, suggesting the presence of a Warburg effect. Under hypoxic conditions, tumor cells may suppress mitochondrial oxidative phosphorylation and instead rely on glycolysis or amino acid metabolism to sustain energy production and rapid proliferation [43]. This metabolic profile is often associated with higher malignant potential and poorer clinical prognosis, indicating that CM2 may represent a more aggressive TIME subtype. In contrast, the GE types enriched in CM5 closely matched the predominant cancer cell features observed in *KRAS* co-mutant patients, consistent with its preferential distribution in this driver mutation. CM5 also exhibited significant enrichment of GE5, suggesting that this subtype may be driven by cancer cells with heterogeneous functional states, thereby generating a more complex TIME. Furthermore, GE1, GE5, and GE9 were enriched in *ROS1*-driven NSCLC (Figure S3E), also supporting the resemblance between the TIME of *ROS1*-driven tumors and CM5.

Given the critical role of copy number variations (CNVs) in driving cancer cell heterogeneity, we next characterized CNV profiles across the four key cancer cell states (Figure 3D and Figure S3A,B, Table S8; Methods). Each state exhibited distinct CNV patterns. In GE1, notable amplifications included *PMVK*, which promotes sustained activation of the aberrantly activated β-catenin signaling pathway in cancer [44]; the proto-oncogene *PBXIP1* [45]; and *PYGO2*, which has been implicated in immune suppression and malignant progression in prostate cancer [46], liver cancer [47], and colorectal cancer [48]. In GE5, *PFKFB2* amplification may inhibit NSCLC invasion and glycolysis via the PI3K/AKT pathway [49], thereby shifting the metabolic preference toward ATP production. Of particular note, *MET* was significantly amplified in GE8, activating the receptor tyrosine kinase c-MET to promote cancer cell migration, invasion, angiogenesis, and EMT [50]. In contrast, GE9 primarily exhibited amplification of calcium-binding proteins such as *S100A2/4/6*, with increased *S100A4* expression being strongly associated with high metastatic potential and poor prognosis [51].

To systematically evaluate the potential malignancy of the five TIME subtypes at the patient level, we conducted survival analyses (Figure 3E, Methods). Among them, CM2 and CM5 were significantly associated with worse patient outcomes, consistent with the cancer cell state profiles enriched in these subtypes. In contrast, CM3 was linked to a favorable prognosis, indicative of a TIME characterized by lower malignant potential and enhanced immune features. Collectively, cancer cell state heterogeneity is tightly associated with the functional characteristics of TIME subtypes, profoundly influencing patient outcomes and providing a theoretical basis for optimizing tumor immunotherapeutic strategies.

### 2.4. Potential Mediators Underlying the Poor-Prognosis CM2 and CM5

**Figure 4:**
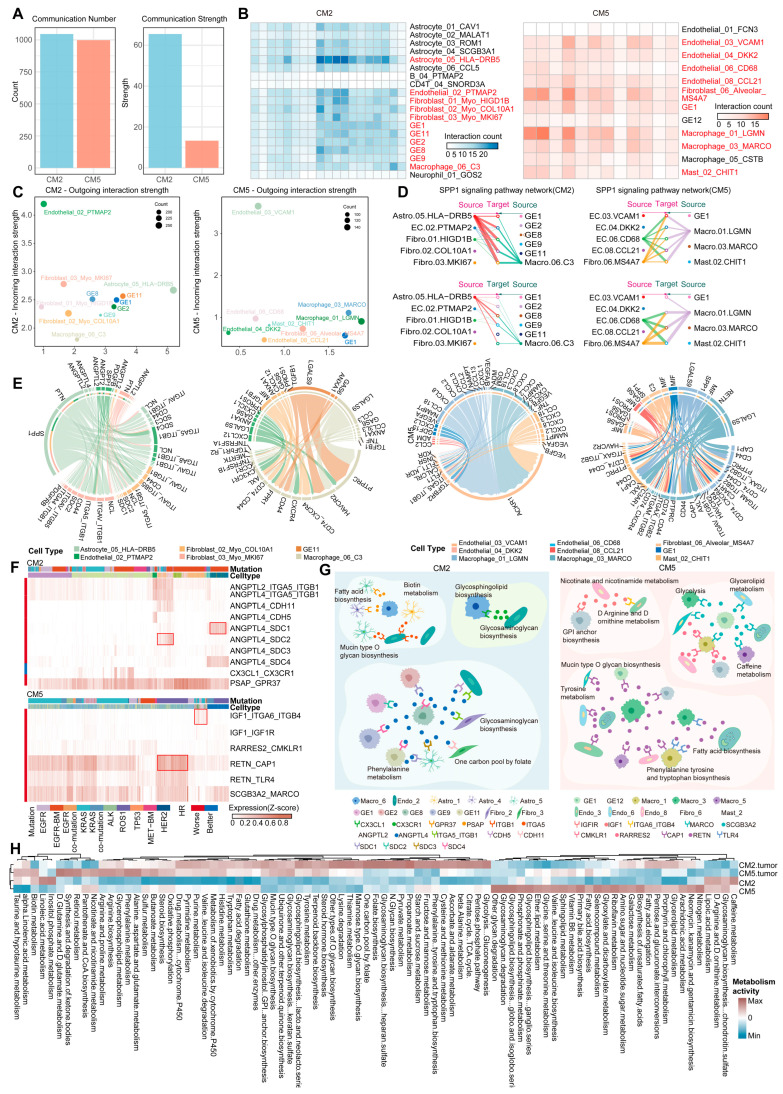
Dissection of potential mediators driving poor prognosis in cellular module (CM)2 and CM5. (**A**) Bar plots showing the number (left) and strength (right) of cell–cell interactions mediated by the CM2 and CM5 subtypes and their associated GEs clusters. (**B**) Heatmaps showing the number of cell–cell interactions for each cellular subcluster within the CM2 and CM5 and their associated GEs clusters. (**C**) Scatter plots showing the outgoing and incoming interaction strength of each cell type in CM2 and CM5. Dot size reflects the number of interactions; color indicates cell identity. (**D**) SPP1 signaling pathways and their corresponding communication networks within CM2- and CM5-associated GEs clusters. Arrows represent directional ligand–receptor interactions among cell types. (**E**) Circo plots showing receptor-ligand pairs involved in key signaling pathways mediated by CM2 (left) and CM5 (right) subtypes. (**F**) Heatmaps showing the expression of HR-related receptor-ligand pairs between CM2 (top) and CM5 (bottom) subtypes and their associated GE clusters. Annotation panels indicate the driver mutation types and cell types. (**G**) Schematic diagram illustrating the mechanisms of potential mediators influencing prognosis in the CM2 (left) and CM (right). (**H**) Heatmap showing metabolic pathway activity across major cell groups within CM2, CM5, and their associated GEs clusters. An overview of cell–cell communication in CM5 and their associated GEs clusters.

Cellular crosstalk within the TIME is pivotal for tumor initiation and progression [52]. We next focused on the intercellular communication networks between CM2 or CM5 and their corresponding cancer cells. Comparative analyses showed that CM2 had significantly more and stronger interactions than CM5, suggesting a more intricate communication network. (Figure 4A and Figure S4A,B, Methods). Specifically, CM2 formed a high-intensity communication network comprising Astrocyte_05_HLA-DRB5, cancer cells, myeloid cells, and multiple fibroblast subpopulations (Figure 4B). Among these fibroblast subsets, *HIGD1B*, *COL10A1*, and *MKI67* were highly expressed, indicating pronounced proliferative capacity [53] and extracellular matrix remodeling potential [54], which may provide a supportive microenvironment for tumor growth and immune evasion [55]. In contrast, the CM5-associated communication network was primarily composed of multiple endothelial cell subsets, alveolar fibroblasts, GE1 cells, and myeloid cells (Figure 4C). Notably, vascular cell adhesion molecule *VCAM1* [56] and the chemokine *CCL21* were predominantly expressed by endothelial cells, whereas *CD68* served as a myeloid cell marker, implying that CM5 is more likely involved in vascular activation, macrophage recruitment, and chemokine-mediated NSCLC metastasis [57].

To elucidate the crucial molecular mechanisms underlying these networks, we further examined the signaling crosstalk patterns within the major communication networks of CM2 and CM5 (Figure S4C,D; Tables S9 and S10). CM2 and CM5 exhibited distinct signaling profiles, while both were characterized by elevated SPP1 signaling mediated by different cell populations (Figure 4D). In CM2, SPP1 signals were predominantly emitted by Astrocyte_05_HLA-DRB5 and Macrophage_06_C3, and were received by endothelial and fibroblast subclusters. Notably, the complement component *C3* and its receptor *C3AR1* have been implicated in the progression of human gliomas, correlating with more aggressive disease phenotypes and shorter survival [58]. These findings suggest that CM2 may represent a specific TIME subtype in NSCLC brain metastases, in which C3^+^ macrophages and astrocyte-like cells cooperatively mediate the SPP1 signaling pathway, potentially contributing to poorer prognosis. In contrast, in CM5, SPP1 signaling primarily originated from CD68^+^ endothelial cells, MS4A7^+^ fibroblasts, and LGMN^+^ macrophages, and was largely received between macrophage subsets and CHIT1^+^ mast cells. This intricate signaling network between myeloid and stromal cells may facilitate the establishment of an invasive and pro-metastatic tumor microenvironment, in line with previous findings that SPP1-driven signaling promotes tumor progression [59].

In addition, CM2-specific PTN and PSAP signaling may also serve as critical drivers of its high invasiveness. PTN signals were jointly emitted by Astrocyte_05_HLA-DRB5 and fibroblasts toward multiple GEs cancer cell subclusters, consistent with pro-tumorigenic mechanisms in bladder cancer [60]. PSAP signaling was broadly produced by nearly all key cellular populations within CM2 and ultimately targeted Astrocyte_05_HLA-DRB5, a pattern closely associated with tumor-promoting and metastatic potential [61]. Ligand–receptor network analysis further revealed that SPP1, the *SDC* gene family, multiple chemokines, and angiopoietin-like protein ANGPTL formed central interaction hubs, collectively contributing to tumor progression (Figure 4E). By comparison, CM5 exhibited activation of MIF, GALECTIN, and RESISTIN pathways, mainly originating from GE1 and multiple other cell types, which targeted LGMN^+^ and MARCO^+^ macrophages as well as mast cells. Previous studies have shown that in TLS-deficient microenvironments, MIF and GALECTIN pathways enhance tumor–immune cell interactions, facilitating the recruitment of immunosuppressive cells and establishing an immunosuppressive niche [62]. Furthermore, BAFF, GALECTIN, and MK signaling axes have been implicated in the induction of immunosuppressive states [63]. We also observed markedly elevated *CXCL* and *CCL* chemokine signaling from *LGMN*^+^ and *MARCO*^+^ macrophages to *VCAM1*^+^ endothelial cells, potentially underlying the pro-angiogenic mechanisms of this subtype. Collectively, the coordinated activation of multiple chemotactic and immunosuppressive signaling pathways in CM5 may drive its immunosuppressive phenotype.

To further delineate the potential mediators of poor prognosis, we assessed the prognostic relevance of subtype-specific ligand–receptor pairs in the two aggressive TIME subtypes, CM2 and CM5. Hazard ratio (HR) analyses identified 10 CM2-specific and 6 CM5-specific ligand–receptor pairs significantly associated with patient prognosis (Figure 4F, Methods). In CM2, *ANGPTL2* is known to promote lymphangiogenesis and plays an important role in lung cancer metastasis [64]. Its mediated ligand–receptor pair was predominantly expressed in fibroblasts from *EGFR*-BM, *KRAS*-mutant, and *KRAS* co-mutant NSCLC, indicating this molecular mechanism may enhance the metastatic potential of lung cancer. *ANGPTL4* has been reported to be expressed in hypoxic NSCLC cells [65] and is associated with the regulation of glutamine metabolism, suggesting its potential involvement in the metabolic reprogramming of cancer cells within CM2. Notably, *ANGPTL4*_*SDC1*/*SDC4* was highly expressed in GE1 cancer cells from *EGFR*-BM, further supporting its role in cancer cell metabolic reprogramming. Additionally, *PSAP*_*GPR37* exhibited consistently high expression across NSCLC driven by different oncogenic mutations, potentially representing a shared mediator of poor prognosis. In contrast, *CX3CL1*_*CX3CR1*, which was associated with a favorable prognosis, showed high expression in Astrocyte_03_ROM1 from *EGFR*-BM. This finding suggests that multiple ligand–receptor mechanisms may cooperatively influence prognosis in this subtype.

In CM5, all six key ligand–receptor pairs were significantly linked to poor prognosis. Among them, *IGF1*_*ITGA6*_*ITGB4*, mediated by endothelial cells and macrophages, emerged as a critical adverse prognostic axis across NSCLC harboring *KRAS*, *EGFR*, and other driver mutations. Previous studies have also implicated *ITGA6* and *ITGB4* in the progression of hepatocellular carcinoma and nasopharyngeal carcinoma [66,67]. *RETN*-mediated ligand–receptor pairs showed a distribution pattern similar to *SCGB3A2*_*MARCO*, with high expression in macrophages from *ROS1*- and *KRAS*-mutant subtypes but low expression in those from *EGFR*-BM NSCLC, suggesting a mutation-subtype-specific mechanism of immune evasion and tumorigenic potential.

Finally, we examined the major metabolic characteristics of the aggressive TIME subtypes and their associated cellular populations (Figure 4H and Figure S4E, Methods). Overall metabolic activity was lower in CM2 than in CM5, whereas CM2-associated cancer cells showed higher activity. Cancer cells from both subtypes were enriched in glycosphingolipid and glycosaminoglycan metabolism, pathways previously linked to cancer cell invasiveness [68], survival, and metastasis [69], suggesting shared metabolic drivers of tumor progression. Notably, CM2-specific activity was enriched in linoleic acid and caffeine metabolism, which have been associated with enhanced T cell function and antitumor immunity, while CM5 exhibited broad metabolic activation, indicating multiple routes to sustain malignancy. Subcluster-level analysis showed that cells of the same type shared metabolic patterns but varied markedly in activity levels; among them, GE9 cancer cells displayed the highest activity, potentially enhancing humoral immune responses. Collectively, these analyses identified key mediators in the two aggressive TIME subtypes that are likely to drive poor prognosis (Figure 4G), providing a potential biological basis for understanding the malignant evolution of the TIME.

### 2.5. Identifying the Driver Mutation Specific Prognostic Signature (DMSP.sig) with Immune Characteristics

**Figure 5:**
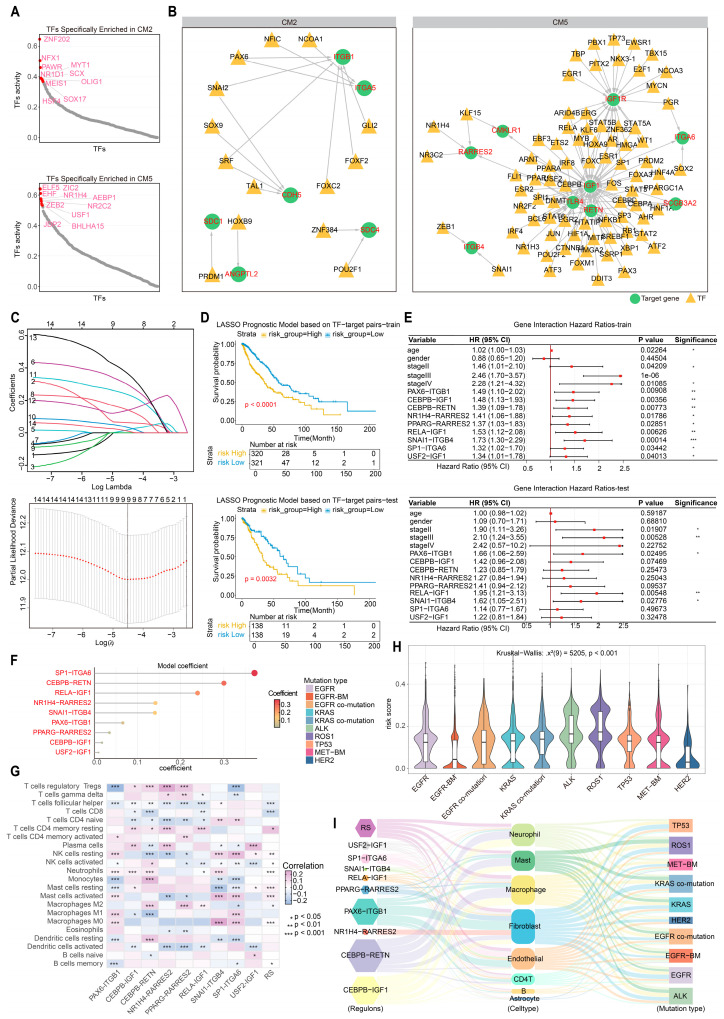
Discovery and functional profiling of the Driver mutation specific prognostic signature (DMSP.sig). (**A**) Scatter plots showing high active TFs in CM2 (top) and CM5 (bottom). The top 10 TFs are highlighted in red. (**B**) Malignant regulatory networks within CM2 (left) and CM5 (right). Circular nodes indicate HR-related ligand/receptor genes, and triangular nodes denote TIME-specific highly active TFs. (**C**) Illustration of the LASSO coefficient spectrum for the overlapping genes (left) and the adjusted parameters of the LASSO regression model (right). (**D**) Kaplan–Meier analysis showing the association between model risk score and OS of patients in the training set (top) and validation set (bottom). Patients were stratified into high-risk and low-risk groups based on the median risk score in both the training set (high-risk: n = 320, low-risk: n = 321) and the validation set (high-risk: n = 138, low-risk: n = 138). Statistical significance was assessed using the log-rank test. (**E**) Multivariable analysis of clinical factors (age, sex, etc.) and the nine constituent regulators as risk factors for overall survival in the training cohort (top) and validation cohort (bottom) The number of asterisks denotes statistical significance levels (* *p* < 0.05, ** *p* < 0.01, *** *p* < 0.001). (**F**) Lollipop plot showing the coefficient values of the 9 regulators in the model. (**G**) The heatmap illustrating the correlations between the model risk score and the nine included regulators with immune cell infiltration, calculated using Pearson’s correlation. (**H**) Violin plots illustrating the distribution of model-derived risk scores across driver-mutated NSCLC samples. Differences among groups were evaluated using the Kruskal–Wallis test. The sample sizes for each subtype are as follows: *EGFR* (n = 11), *EGFR-BM* (n = 10), *EGFR* co-mutation (n = 2), *KRAS* (n = 12), *KRAS* co-mutation (n = 3), *ROS1* (n = 3), *TP53* (n = 1), *ALK* (n = 1), *MET* brain metastasis (*MET*-BM, n = 1), and *HER2* (n = 1). (**I**) Sankey diagram illustrating the associations of the model-derived risk score and its nine constituent regulators with cell types and driver mutation subtypes of NSCLC. The width of each flow represents the strength of the association.

Dysregulation of transcription factor (TF) expression or activity can disrupt cellular homeostasis, thereby driving tumor initiation and progression [70]. To investigate mechanisms of homeostatic imbalance and immune remodeling in the aggressive TIME subtypes CM2 and CM5, we assessed their key TF activities (Figure 5A, Methods). In CM2, *ZNF202* and *NFX1* exhibited marked activation. *ZNF202* has previously been identified as a poor prognostic factor in head and neck squamous cell carcinoma [71]. It regulates cholesterol efflux by modulating *ABCA1* expression through the macrophage-specific TLR2/NF-κB/ZNF202 signaling axis [72]. *NFX1* may promote immune evasion by suppressing the expression of its target MHC class II genes and modulating inflammatory responses [73]. Thus, CM2 may be driven by macrophage-centered metabolic reprogramming and impaired antigen presentation, together contributing to its immunosuppressive phenotype and poor outcome. In contrast, the pronounced activation of epithelial-specific TES TFs *ELF5* and *EHF* in CM5 may jointly facilitate NSCLC progression [74] and regulate angiogenesis [75].

To delineate the malignant regulatory programs underlying the aggressive TIME subtypes, we constructed subtype-specific regulatory networks linking active TFs to HR-related ligand–receptor genes (Methods). These networks focus on the regulation of HR-related ligand–receptor pairs within core interacting cells, indicating that these transcription factors may drive immunosuppression and tumor progression (Figure 5B). HR analyses further identified core regulons associated with patient risk, supporting their key roles in the malignant evolution of aggressive TIME subtypes (Table S11). In parallel, we characterized the key CNV and driver gene–associated regulons in malignant cells associated with each subtype (Figure S5A,B). Of note, while CM5 exhibited a more intricate malignant regulatory network involving angiogenesis and metabolic reprogramming processes mediated by multiple regulons such as *CMKLR1* [76,77], the regulatory networks within CM2-associated cancer cells appeared even more complex than those in CM5, implying a stronger engagement of malignant driving forces.

**Figure 6:**
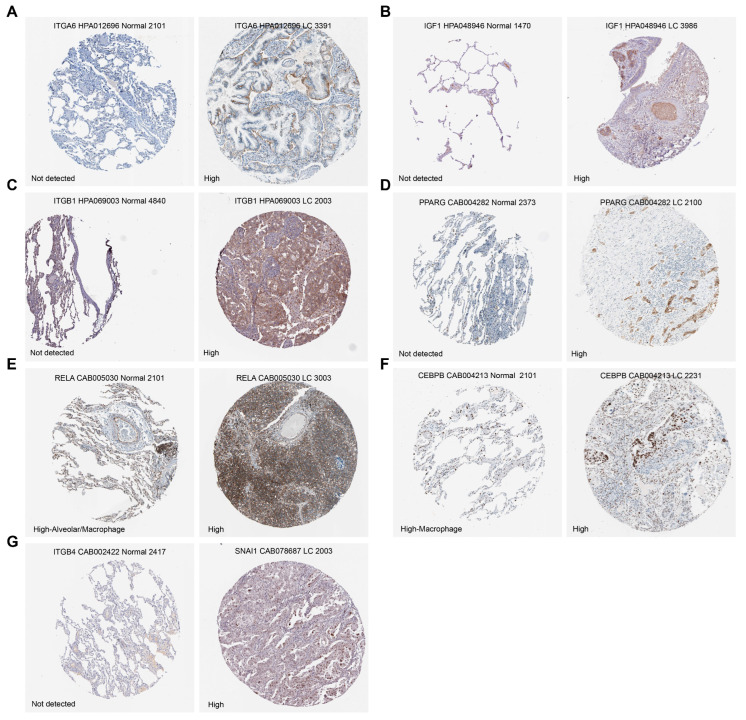
Immunohistochemical staining for modulators of the prognostic model. (**A**–**F**) IHC staining of *ITGA6*, *IGF1*, *ITGB1*, *PPARG*, *RELA* and *CEBPB* in normal lung tissue and lung cancer tissue. The protein-level expression patterns of these model modulators were validated, showing elevated expression of selected genes or transcription factors in tumor samples compared with normal tissues. (**G**) IHC staining of *ITGB4* and *SNAI1* in normal lung tissue and lung cancer tissue. Consistently, *ITGB4* protein expression was not detected in normal samples, while *SNAI1* exhibited markedly higher expression in tumor tissues. All representative staining images in panels (**A**–**G**) were obtained from the publicly available Human Protein Atlas (HPA) database. Each tissue section is annotated at the top with the gene/transcription factor name, antigen type, tissue type, and patient ID. The expression level of the corresponding protein is indicated at the bottom left. Scale bar = 200 µm. Each tissue core has a diameter of approximately 1 mm.

Given the close association of these malignant regulons with patient prognosis and their potential roles in mediating immunosuppression and tumor progression, we further integrated those with significant prognostic value to construct a prognostic scoring model based on their expression features, aiming to systematically evaluate its predictive performance in NSCLC risk stratification (Methods). Specifically, we utilized the TCGAplot package to access bulk RNA-seq data from 917 NSCLC patients and randomly divided the cohort into training and validation sets at a 7:3 ratio (Methods). In the training cohort, univariate Cox regression of 63 HR-related candidate regulons identified 14 high-risk regulons. We then applied LASSO regression with 10-fold cross-validation to minimize overfitting and collinearity, yielding nine TF–ligand–receptor regulons (*PAX6*–*ITGB1*, *CEBPB*–*IGF1*, *CEBPB*–*RETN*, *NR1H4*–*RARRES2*, *PPARG*–*RARRES2*, *RELA*–*IGF1*, *SNAI1*–*ITGB4*, *SP1*–*ITGA6*, and *USF2*–*IGF1*). The final Risk Score (RS) was calculated as the sum of these regulons weighted by their respective coefficients (Figure 5C; Methods; Table S12). To further provide orthogonal validation at the spatial level, we analyzed an independent spatial transcriptomics dataset (E-MTAB-13530) comprising tumor and adjacent non-tumor tissues from NSCLC patients. The identified prognostic regulons (transcription factor–target gene pairs) exhibited tumor-specific co-localization patterns to varying degrees across patients. Among them, six regulon pairs showed robust tumor-specific co-localization signals in multiple patients. Notably, in patients P16 and P19, all six regulon pairs—including *USF2*–*IGF1*, *RELA*–*IGF1*, and *SP1*–*ITGA6*—displayed pronounced co-localization specifically within tumor regions. Furthermore, key regulons such as *USF2*–*IGF1*, *RELA*–*IGF1*, and *SNAI1*–*ITGB4* were consistently highly expressed in tumor regions across five patients, suggesting that these transcriptional regulatory relationships may represent shared tumor-promoting mechanisms across patients. Collectively, these findings provide spatial evidence supporting the robustness of our prognostic model (Figure S7A). Furthermore, we assessed the protein expression of transcription factors and genes within these nine regulons from the publicly available Human Protein Atlas (HPA) database (http://www.proteinatlas.org, accessed on 4 December 2025) for normal and lung cancer tissues. Notably, *ITGA6*, *IGF1*, *ITGB1*, and *PPARG* displayed tumor-specific upregulation, with little to no expression detected in normal tissues, underscoring their functional relevance in the malignant context (Figure 6A–G; Methods).

To evaluate model performance, patients in the training and validation cohorts were stratified into high- and low-risk groups using the median RS. Model performance was assessed by Kaplan–Meier survival analysis (Methods), ROC curves, and the RS distributions. Kaplan–Meier analysis demonstrated significantly longer survival in the low-risk group in both cohorts (Figure 5D). Furthermore, subgroup analyses stratified by histological subtype (LUAD and LUSC) and clinical stage (I–IV) consistently showed poorer survival in the high-risk group, particularly in LUAD, LUSC, and early-stage patients in the training cohort. In the validation cohort, a similar trend was observed, with LUAD and stage II patients in the high-risk group exhibiting worse survival outcomes, supporting the robustness of the model across different clinical contexts (Figure S6A–L). ROC analysis confirmed the model’s discriminative capacity for overall survival (training cohort AUCs at 1, 3, and 5 years: 0.68, 0.65, 0.66; validation cohort: 0.54, 0.64, 0.69) (Figure S5C), and high-risk scores were consistently associated with increased mortality (Figure S5D). Assessment of clinical factors revealed that patients with stage II or III disease had significantly worse outcomes in both cohorts, and risk increased with tumor stage (Figure 5E). Among the regulons, *RELA*–*IGF1* and *SNAI1*–*ITGB4* were robustly associated with poor prognosis in both cohorts, ranking third and fifth, respectively, by coefficient magnitude in the model (Figure 5F). The top contributor, *SP1*–*ITGA6*, also showed strong oncogenic features; *SP1* has been shown to enhance tumor resistance to programmed cell death and promote immune evasion [78], while *ITGA6* can drive radio resistance in NSCLC via the ITGA6/PI3K/Akt signaling axis [79], suggesting a cooperative role in supporting tumor cell survival and therapy resistance in NSCLC.

To investigate the association between the risk model and immune landscape shaping, we examined the correlations between the risk scores, the nine constituent regulons, and immune cell infiltration levels (Figure 5G, Methods). Notably, high-risk scores were associated with reduced infiltration of CD8^+^ T cells and helper T cells, increased infiltration of dormant memory T cells, and high infiltration of both activated and resting mast cells. These patterns suggest that high-risk patients reside in a microenvironment with impaired immune defense and sustained pro-tumor inflammatory signaling. Further analyses revealed that several key regulons exhibited consistent associations with reduced infiltration of anti-tumor immune populations, including helper T cells [80] and activated dendritic cells (DC) [81]. Conversely, certain regulons had divergent effects: *PPARG*–*RARRES2* and *CEBPB*–*RETN* were positively correlated with high infiltration of pro-tumor, anti-inflammatory M2 macrophages [82], whereas *SP1*–*ITGA6* was associated with low infiltration of M2 macrophages. By weighting each regulon’s expression with its corresponding coefficient, the model captures a composite immunological state characterized by a “desert” of immune-activating cells alongside activation of tumor-promoting cells.

Finally, we systematically examined the association between the risk model and NSCLC driver mutation subtypes (Figure 5H). The RS varied significantly across subtypes: patients with *ALK* or *ROS1* alterations generally showed higher scores, whereas those with *EGFR*-BM or *HER2* mutations had lower scores. These differences suggest that the RS captures essential distinctions in tumor progression patterns and immune microenvironmental features among driver mutation subtypes, offering insights into subtype-specific mechanisms and potential therapeutic targets. On this basis, we designated the model as the Driver mutation specific prognostic signature (DMSP.sig). We further evaluated score distributions across subtype-related cellular modules (Figure 5I; Figure S5E). CM2, representing a brain metastasis–associated microenvironment dominated by astrocytes and diverse stromal cells, had consistently lower scores. In contrast, CM5, largely corresponding to *ROS1*-, *KRAS*-, and *ALK*-driven tumors, exhibited a macrophage-enriched TIME with markedly elevated scores, driven predominantly by *CEBPB*–*RETN* and *CEBPB*–*IGF1*. Taken together, the DMSP.sig not only provides robust prognostic prediction in NSCLC but also delineates striking differences in immune composition and malignant regulatory programs across driver mutation subtypes. The model captures a dysregulated immune contexture, defined by the “desertification” of immune-activated cells alongside the activation of pro-tumor cells, thereby offering a framework for molecular subtyping and precision therapeutic strategies.

### 2.6. Identification of Potential Therapeutic Agents Targeting Prognostic Biomarkers

**Figure 7:**
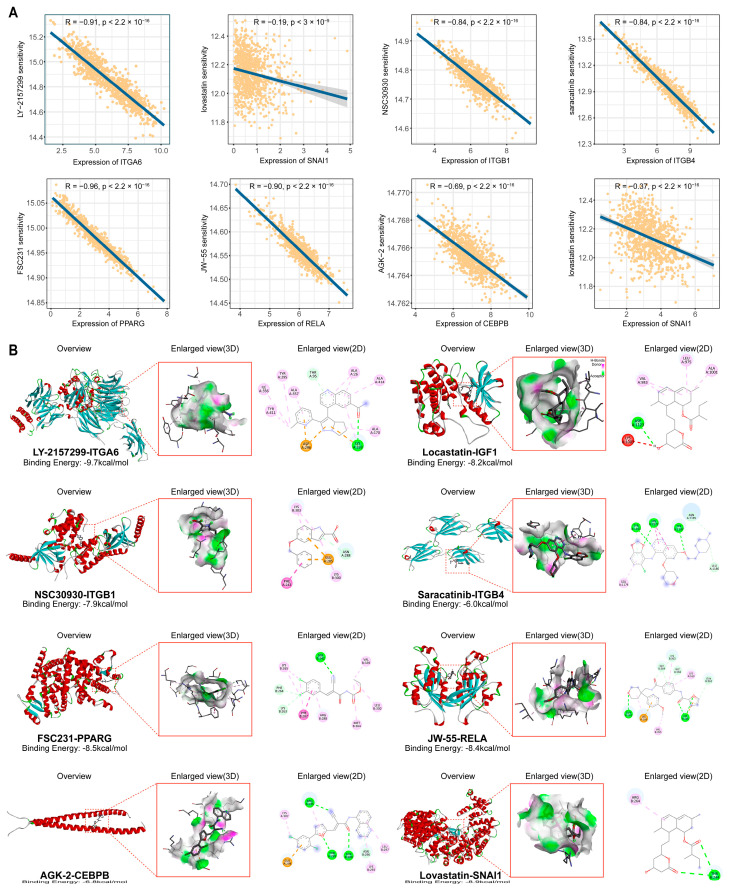
Drug sensitivity and molecular docking analyses. (**A**) Scatter plots illustrating the correlations between target gene expression levels and predicted drug sensitivities, with trend lines indicating the overall strength of the associations. All identified associations exhibit significant negative correlations (R < 0), indicating that higher gene expression levels correspond to increased drug sensitivity (lower IC50). (**B**) The molecular docking results between target drugs and core targets. The binding energy (kcal/mol) for each target protein–drug pair is indicated, with lower values representing stronger binding affinity, facilitating direct comparison of docking performance across different compounds.

Given the close association between the identified DMSP.sig and patient prognosis as well as immune status within the tumor microenvironment, we next sought to identify potential therapeutic agents that may target these prognostic markers. Based on a subset of tumor-specific DMSP.sig markers that were validated at the protein level, drug sensitivity analysis was performed (Methods). Using oncoPredict, we identified multiple compounds exhibiting high predicted sensitivity across these eight prognostic biomarkers (Table S13). For each target gene, the compound with the highest predicted sensitivity was further highlighted (Figure 7A). Overall, the predicted sensitivities of the identified compounds showed significant positive correlations with the expression levels of all eight prognostic genes. Notably, the predicted sensitivities of LY-2157299, FSC231, and JW-55 exhibited particularly strong correlations with the expression of *ITGA6*, *PPARG*, and *RELA*, respectively, with correlation coefficients less than −0.90. These results indicate that higher expression of these prognostic markers is associated with increased drug sensitivity, suggesting that these compounds may exert therapeutic effects by targeting pathways related to these genes, thereby potentially modulating immune-related phenotypes and contributing to prognostic improvement.

To further evaluate the binding capacity between candidate drugs and their corresponding target proteins, molecular docking analyses were conducted using protein structural models and small-molecule conformations (Figure 7B, Methods). The results showed that the binding energies of all drug–target complexes were lower than −5 kcal/mol, indicating favorable binding affinity. In particular, LY-2157299 exhibited a strong binding interaction with the protein encoded by *ITGA6*, with a binding energy of −9.7 kcal/mol. This finding is consistent with the predicted drug sensitivity results, further supporting the strong interaction between LY-2157299 and *ITGA6*, and suggesting that this compound may effectively interfere with *ITGA6*-associated intercellular interactions, thereby contributing to improved patient prognosis.

## 3. Discussion

In this study, we systematically analyzed NSCLC harboring driver mutations, extending the focus from histopathology to molecular subtypes to delineate immune heterogeneity. Previous studies have shown that driver-mutant NSCLC requires distinct therapeutic strategies and exhibits variable responses to immune checkpoint inhibitors (ICIs) [83]. However, most single-cell studies have focused on LUAD and LUSC [20] or concentrated on single mutations such as *EGFR* [84,85], leaving the immune heterogeneity among multiple driver subtypes insufficiently characterized. In contrast to previous reports describing mixed-lineage phenotypes across NSCLC, our study systematically links specific driver mutations to distinct immune microenvironmental states [86]. However, these studies largely lacked a systematic comparison across multiple oncogenic drivers within a unified analytical framework, limiting cross-mutational interpretability.

To address this gap, we integrated approximately 200,000 scRNA-seq profiles and systematically mapped the TIME landscape in NSCLC with diverse driver backgrounds. Compared with earlier studies that stratified LUAD by radiological features such as ground-glass nodule (GGN) and part-solid nodule (PSN), our study shifts toward identifying mutation-associated TIME subtypes. While prior work focused on cell-level immune dynamics, our analysis captures coordinated changes at the level of TIME subtypes [87]. We further incorporated the contributions of stromal cells to tumor invasion and identified five CMs (Table S14), representing functionally distinct TIME states that more precisely capture the immune features of driver-mutant NSCLC. Notably, CM1 closely resembled the previously reported immune-activated TIME (Figure S2B), predominantly composed of lymphocyte subsets. In contrast, CM2 and CM5 exhibited immunosuppressive and tumor-promoting features, with CM5 resembling immune-excluded TIME but differing in cellular composition. Moreover, these TIME subtypes displayed subtype-specific distributions: CM1 and CM3 were enriched in *EGFR*-mutant NSCLC, CM2 was enriched in *EGFR*-mutant brain metastases, CM4 in *KRAS*-mutant NSCLC, and CM5 in *ROS1*/*ALK*-rearranged NSCLC. Importantly, *EGFR*-mutant NSCLC showed altered TIME phenotypes upon brain metastasis, consistent with previous reports.

Given the pronounced differences in malignant pathway activity across cell programs, we further explored associated cancer cell states. Unlike conventional clustering analyses, our study captured both intra- and intertumoral molecular features, identifying 13 functionally distinct GEs (Table S14). Among these, GE1, GE5, GE8, and GE9 were significantly associated with TIME programs, supporting prior evidence that oncogenic pathways modulate immune composition [32,88]. Specifically, CM2 was characterized by reduced expression of oxidative phosphorylation–related GE1, reflecting a Warburg-like metabolic profile that may account for its invasiveness and immunosuppressive properties [89]. In contrast, CM5 exhibited broad activation of *EGFR*, WNT, and JAK-STAT pathways and was closely linked to key malignant states, indicating complex tumor–immune interactions. Mechanistically, CM2 was defined by astrocyte–fibroblast–C3^+^ macrophage axes (SPP1, PTN, PSAP) mediating immunosuppression and promoting *EGFR*- and *MET*-associated brain metastasis, reminiscent of immunosuppressive mechanisms observed in retinoblastoma. CM5, however, relied primarily on myeloid–endothelial interactions, chemokine activation, and stromal signaling, shaping the immunosuppressive TIME in *ROS1*-, *KRAS*-, and *ALK*-mutant tumors. Previous ligand–receptor-based studies of intercellular communication in NSCLC are largely limited to static interaction maps and fail to capture upstream transcriptional regulators [90]. In contrast, our analysis extends beyond ligand–receptor interactions by identifying transcription factor-driven regulatory programs that orchestrate intercellular signaling networks. Collectively, these findings provide a comprehensive view of mutation specific molecular mechanisms underlying invasive TIME subtypes.

Building on these observations, we identified the DMSP.sig. Unlike previous NSCLC prognostic models [91,92,93], which are predominantly based on bulk transcriptomic signatures or differentially expressed genes and do not explicitly incorporate tumor microenvironment states or cell–cell communication architectures, this model was derived from invasive subtypes CM2 and CM5, integrating key ligand–receptor pairs and high-risk TFs to capture dual immune states of desertification and tumor-promoting activation quantitatively. Risk scores varied significantly across driver subtypes, reflecting the mutation specific contribution to TIME remodeling and providing a robust predictor of patient prognosis. This framework highlights the interplay among driver mutations, malignant pathways, and immunosuppressive networks, offering insights into NSCLC immune evolution and potential therapeutic targets. In addition, the stratification defined by DMSP.sig provided a practical framework for prioritizing drugs with mutation- and microenvironment-specific sensitivity, linking prognostic risk states to therapeutic vulnerability.

Several limitations should be acknowledged. First, rare driver subtypes were underrepresented due to their low incidence, which may limit the generalizability of our findings. Second, the study primarily focused on invasive subtypes CM2 and CM5, whereas the favorable CM3 subtype was not extensively explored; its molecular basis warrants validation in larger cohorts and functional studies. Our study provides key insights into TIME heterogeneity in driver-mutant NSCLC and lays a foundation for precision immunotherapy. Future studies integrating larger datasets and multi-omics analyses are expected to further enhance the robustness of these findings and facilitate their clinical translation.

## 4. Materials and Methods

### 4.1. Sample Characteristics

To construct a comprehensive single-cell transcriptomic atlas encompassing multiple driver mutations of NSCLC, we integrated scRNA-seq data from 45 untreated patients with driver mutation–positive NSCLC, collected from seven publicly available datasets. Based on gene mutation type (e.g., *EGFR*, *KRAS*), mutation pattern (single driver mutation or co-mutation), and tissue source (primary tumor or brain metastasis), patients were classified into ten distinct driver mutation subtypes: *EGFR* (n = 11), *EGFR*-BM (n = 10), *EGFR* co-mutation (n = 2), *KRAS* (n = 12), *KRAS* co-mutation (n = 3), *ROS1* (n = 3), *TP53* (n = 1), *ALK* (n = 1), *MET* brain metastasis (*MET*-BM, n = 1), and *HER2* (n = 1) (Table S1).

Bulk RNA-seq data for TIME subtype survival analyses were retrieved from The Cancer Genome Atlas (TCGA; National Cancer Institute and National Human Genome Research Institute, Bethesda, MD, USA), comprising 200 lung adenocarcinoma (LUAD) and 200 lung squamous cell carcinoma (LUSC) samples; after quality control, 386 cases were retained. For prognostic model training and validation, bulk RNA-seq data were obtained from the LUAD and LUSC datasets embedded in the TCGAplot package (v8.0.0), totaling 917 patients, and randomly split into a training cohort (n = 641) and a validation cohort (n = 276) in a 7:3 ratio.

### 4.2. Processing and Annotation of scRNA-seq Data

To ensure uniform gene nomenclature across datasets, gene names were converted to the “SYMBOL” format using the R package org.Mm.eg.db (v3.20.0; Bioconductor, Boston, MA, USA). Given the dataset-specific distributions of UMI counts, gene features, and mitochondrial gene percentages, quality control was performed separately for each dataset. Low-quality cells were removed if they had UMI counts ≤ 50,000, ≤200 detected genes, or ≥25% mitochondrial gene content. Highly variable genes (top 2000) were identified using the FindVariableFeatures function, followed by principal component analysis (PCA) for dimensionality reduction. Batch effects were corrected using Harmony (v1.2.3, Broad Institute of MIT and Harvard, Cambridge, MA, USA).

Based on the Harmony output, clustering was performed with Seurat (v5.3.0; Satija Lab, New York Genome Center, New York, NY, USA). a clustering tree was constructed using the Clustree package (v0.5.1; CRAN, R Foundation for Statistical Computing, Vienna, Austria), with an optimal resolution of 0.4, resulting in 23 clusters. Marker genes for each cluster were identified with FindAllMarkers.

Cell type annotation combined predictions from SingleR (v2.8.0; Bioconductor, Boston, MA, USA) with top 20 cluster-specific DEGs matched to the CellMarker 2.0 database (College of Bioinformatics Science and Technology, Harbin Medical University, Harbin, China), further refined by known lineage markers. Annotation accuracy was confirmed through differential expression analysis across cell types.

Major TIME populations, including myeloid cells, B cells, T cells, and stromal cells, were subjected to secondary clustering. Optimal resolutions of 0.6, 0.4, 0.5, and 0.2 were selected based on hierarchical relationships, and subclusters were annotated by integrating their DEGs with canonical markers and functional features, e.g., Macrophage_01_LGMN within the myeloid compartment.

### 4.3. Immune Composition Analysis

To assess the immune composition characteristics of NSCLC subtypes with different driver mutations, we referenced the immune cell classification method from existing literature [15], where innate immunity is primarily mediated by myeloid cells, and adaptive immunity involves T cells and B cells. The overall proportion of myeloid cells was calculated for each subtype, along with the overall proportion of T cells and B cells, followed by normalization. The *t*-test was then used to assess the statistical significance of distribution differences between subtypes with different driver mutations.

### 4.4. Ro/e Ratio Analysis

In NSCLC samples with different driver mutations, the ratio of observed cell counts to the expected cell counts, based on the overall distribution (Ro/e), was calculated for the 64 cell subpopulations in the TIME to characterize the tissue preference of each subpopulation. A Ro/e value greater than 1 indicates that the cell subpopulation shows tissue preference in that driver mutation subtype, and statistical significance was assessed using the Chi-square test.

Similarly, to compare the distribution differences in the five TIME subtypes across NSCLC samples with different driver mutations, we calculated the Ro/e values for each TIME subtype in different driver mutation NSCLC subtypes and determined tissue preference and significance levels according to the same criteria.

### 4.5. Cell Module Identification

To dissect immune states within the TIME of driver mutation–positive NSCLC, a co-occurrence analysis was performed for the 64 TIME subpopulations. The procedure is as follows: first, the normalized frequency of each cell subpopulation in patient samples was calculated, excluding unclassified “unknown” cells. Next, the pairwise Pearson correlation coefficients between the normalized frequencies of cell subpopulations were calculated using the corr. test function from the R package psych (v2.5.6; CRAN, R Foundation for Statistical Computing, Vienna, Austria), with statistical significance assessed via a *t*-test. Based on the correlation matrix, hierarchical clustering of cell subpopulations (ward.D2 method) and visualization were performed using the R package pheatmap (v1.0.13; CRAN, R Foundation for Statistical Computing, Vienna, Austria). Ultimately, five stable cell modules were identified, each representing a co-existing or functionally similar TIME subtype in patients.

### 4.6. Differential Gene Expression and Pathway Enrichment Analysis

For each TIME subtype, the top 200 differentially expressed genes (DEGs) were subjected to Gene Ontology (GO) biological process (BP) enrichment analysis using the enricherGO function of the clusterProfiler package (v4.14.6; Bioconductor Project, Buffalo, NY, USA) with parameters pvalueCutoff = 0.05 and qvalueCutoff = 0.2. The top 10 enriched pathways were visualized.

Functional comparisons among TIME subtypes were further conducted via KEGG pathway enrichment using the compareCluster function (fun = “enrichKEGG”), based on the KEGG database (Kanehisa Laboratories, Kyoto, Japan), with visualization of the top five pathways for each subtype.

### 4.7. Gene Set Scoring

Based on 24 published immune-related gene sets, we used the AddModuleScore function from the R package Seurat to score each single cell in the TIME, calculating the average score for all cells within each TIME subtype, and used the Kruskal–Wallis test to assess the statistical significance of the differences.

To assess the GEs status of cancer cells, we also used the AddModuleScore function, scoring the cancer cells identified by CopyKat using 13 GEs gene sets and defining GEs labels for each cancer cell.

To analyze the association between the 13 cancer cell GEs and the TIME subtypes, as well as NSCLC subtypes with different driver mutations, the AddModuleScore function was again applied to score the single cells, calculating the average score for all cells in each TIME subtype and each driver mutation subtype. Statistical significance was assessed using analysis of variance (ANOVA).

### 4.8. Determination of Dominant Cellular Modules in Patients

For each NSCLC patient, the normalized frequency of cell clusters originating from the same TIME subtype was calculated. The summed frequencies across the five TIME subtypes were subsequently normalized, and the subtype with the highest frequency was designated as the dominant TIME type for the patient.

### 4.9. Cancer Cell State Identification

Raw counts of epithelial cells were analyzed for CNVs using CopyKat (v1.0.8; Navin Laboratory, MD Anderson Cancer Center, Houston, TX, USA), with a minimum of five genes per chromosome and clustering based on Euclidean distance. Cells were classified into malignant and non-malignant subpopulations according to their CNV patterns, with malignant epithelial cells defined as cancer cells. The cancer cells were preprocessed using NormalizeData, FindVariableFeatures, and ScaleData functions, and patient samples with fewer than 50 cells were excluded. PCA and dimensionality reduction were subsequently performed. To capture both intra- and inter-tumoral heterogeneity, multi-resolution unsupervised clustering was conducted with resolutions ranging from 0.01 to 2. Upregulated DEGs were identified for clusters at each resolution using FindAllMarkers, and DEGs across all resolutions were merged. Genes with mitochondrial genes, adjusted *p* ≥ 0.05, log2FoldChange ≤ 0, or expressed in ≤20% of cells were excluded. For each cluster, the top 200 marker genes ranked by adjusted *p* value and log2FoldChange were selected. A similarity matrix of cluster marker gene sets was constructed based on the Jaccard index, removing gene sets with fewer than 20 genes and eliminating redundancy with similarity >0.95. Consensus clustering using the R package cola (v2.12.0; Bioconductor Project, Buffalo, NY, USA) was then applied to the deduplicated Jaccard matrix to identify GEs, with ATC as the feature selection method and skmeans as the clustering algorithm. The optimal number of clusters was determined to be 13, resulting in 13 cancer cell state-related GEs.

### 4.10. CNV Level Analysis

To evaluate gene-level CNV in each GEs cancer cell population, infercnv (v1.22.0; Broad Institute of MIT and Harvard, Cambridge, MA, USA) was applied using DC and NK cells as references. CNV matrices of cancer cells identified by CopyKat and genomic annotation were extracted. For cancer cells labeled with GEs, the mean CNV signal of each gene across GEs states was calculated, reflecting amplification (positive values) or deletion (negative values). Genes with the most significant amplification or deletion were selected based on genomic position and visualized using the R package circlize (v0.4.16; Bioconductor Project, Buffalo, NY, USA).

### 4.11. Survival Analysis

To assess the prognostic value of TIME subtypes, the top 30 marker genes identified from single-cell analysis were extracted from bulk RNA-seq of 386 NSCLC patients. The mean expression of each module per patient was calculated as the module score. Optimal cutoffs were determined using surv_cutpoint (survmine v0.5.0; CRAN, R Foundation for Statistical Computing, Vienna, Austria) to classify patients into high-expression and low-expression groups. Kaplan–Meier curves were constructed, survival differences assessed using survfit (survival v3.8-3; CRAN, R Foundation for Statistical Computing, Vienna, Austria), and curves with risk tables plotted via ggsurvplot.

For DMSP.sig, survival analyses were performed separately in the training cohort (641 patients) and validation cohort (276 patients), with median risk scores dividing patients into high- and low-risk groups, followed by Kaplan–Meier analysis to evaluate prognostic performance.

### 4.12. Cellular Interaction Analysis

CellChat (v2.2.0; University of California, Irvine, Irvine, CA, USA) was employed to analyze interactions mediated by invasive subtypes CM2 and CM5, using the CellChatDB v2 ligand-receptor database (University of California, Irvine, Irvine, CA, USA) to assess intercellular communication weights. CM2 and its corresponding cancer cells were merged into a Seurat object, excluding clusters with fewer than 50 cells; the same procedure was applied for CM5. Communication probabilities between clusters were quantified based on the mean expression of ligands, receptors, and cofactors. Network centrality metrics were computed using netAnalysis_computeCentrality, and signaling input/output patterns were visualized with netAnalysis_signalingRole_heatmap. Key ligand-receptor interactions were illustrated using netVisual_chord_gene.

### 4.13. Metabolic Analysis

The R package scMetabolism (v0.2.1; scMetabolism development team, GitHub, Inc., San Francisco, CA, USA) was used to analyze the metabolic pathway activity of the invasive subtypes CM2 and CM5, as well as their corresponding cancer cell populations (CM2.epi and CM5.epi). Based on the KEGG metabolic pathway database (Kanehisa Laboratories, Kyoto, Japan), the AUCell (v1.28.0; Bioconductor Project, Buffalo, NY, USA) algorithm was used to calculate the activity scores of 85 core metabolic pathways to assess the overall metabolic differences in the four groups. Additionally, the activity of the same set of metabolic pathways was further evaluated at the cell cluster level within these groups to reveal the metabolic heterogeneity between cell clusters. Statistical significance was assessed using the non-parametric Mann–Whitney U test, and false discovery rate (FDR) correction was performed using the Benjamini–Hochberg method.

### 4.14. HR Analysis

To assess the prognostic risk of key cell clusters within the 64 TIME subpopulations, the top 10 marker genes for each subgroup (after removing aberrant or redundant gene sets) were used, and gene set scores were calculated for 917 tumor samples from TCGA LUAD and LUSC cohorts using the R package GSVA (v2.0.7; Bioconductor Project, Buffalo, NY, USA). Combined with patient survival data, a Cox proportional hazards regression model (with age as a covariate) was used to calculate the HR and 95% confidence intervals for each gene set in LUAD and LUSC, with statistical significance assessed via Wald tests. Ultimately, all results were classified based on the HR direction into “Worse survival” (HR > 1) and “Better survival” (HR < 1).

For the HR analysis of regulons (TFs and target genes), the same method was applied. A regulon was broken down into gene sets containing 2-3 elements, and their prognostic value was assessed in TCGA LUAD and LUSC.

### 4.15. TF Identification

Key TFs in CM2, CM5, and their corresponding cancer cells were identified using run_ulm (decoupleR v2.12.0; Bioconductor Project, Buffalo, NY, USA), selecting factors with high subtype-specific activity. Chi-square tests (chisq.test) were applied to compare TF specificity between CM2 and CM5, and factors were ranked by chi-square value to determine subtype-specific TFs.

### 4.16. Prognostic Model Construction

A prognostic model for NSCLC was built based on malignant regulatory networks in CM2 and CM5, including TFs and their target genes. GSVA (v2.0.7; Bioconductor Project, Buffalo, NY, USA) computed enrichment scores of 63 regulons across 917 LUAD and LUSC patients, generating a candidate scoring matrix. Samples were randomly split 7:3 into training (n = 641) and validation (n = 276) sets. In the training cohort, univariate Cox regression identified 14 significant regulons (*p* < 0.05), followed by LASSO Cox regression (glmnet v4.1-8; CRAN, R Foundation for Statistical Computing, Vienna, Austria) to select nine regulons and construct the final model.

The final prognostic model (DMSP.sig) was constructed using a weighted linear combination of the enrichment scores for these nine regulons, with the risk score (RS) for each patient calculated as follows: $RS=\sum_{i=1}^{9}{Coefficient}_{i}{\;*\;Regulon\;exp}_{i}$. where Coef*_i_* represents the multivariate Cox regression coefficient and Regulon exp*_i_* represents the GSVA enrichment score of the i-th regulon. The specific coefficients for the nine regulons are detailed in Supplementary Table S1.

RS were calculated for training and validation cohorts, with Kaplan–Meier curves and time-dependent ROC curves assessing 1-, 2-, and 3-year predictive performance. Model risk scores were further correlated with survival status using ggrisk (v1.3; CRAN, R Foundation for Statistical Computing, Vienna, Austria).

### 4.17. Spatial Transcriptomics-Based Regulon Co-Localization Analysis

Spatial transcriptomics data (E-MTAB-13530) were obtained from the EMBL-EBI database, including tumor and adjacent non-tumor samples from eight NSCLC patients. For each sample, spatial transcriptomics objects were constructed using the Load10X_Spatial() function in the Seurat package.

To evaluate the spatial expression characteristics of prognostic regulons (transcription factor–target gene pairs), the SpaGene package (version 0.1.0; SpaGene development team, GitHub, Inc., San Francisco, CA, USA) was applied. Specifically, the SpaGene_LR() function was used to assess both individual gene expression and spatial co-localization patterns of each regulon across tissue sections. Comparative analyses between tumor and adjacent non-tumor regions were performed to identify tumor-specific co-localization patterns of prognostic regulons.

### 4.18. HPA Immunohistochemistry Validation

IHC data were downloaded from the HPA (HPA; Science for Life Laboratory, Stockholm, Sweden; http://www.proteinatlas.org, accessed on 4 December 2025) to evaluate the expression levels (high or low) of the genes or transcription factors contained within the nine regulons in both normal lung tissue and lung cancer tissue.

### 4.19. Immune Infiltration Analysis

Immune infiltration analysis was performed using TCGAplot (v8.0.0; TCGAplot development team, Tongji Hospital, Tongji Medical College, Huazhong University of Science and Technology, Wuhan, China) for 917 LUAD and LUSC samples. Immune cell proportion matrices were derived using get_immu_ratio(), matched with GSVA scores, and expression of nine regulons. Pearson correlation coefficients between regulons/RS and immune cell infiltration were calculated using corr.test(), with significance levels indicated as * *p* < 0.05, ** *p* < 0.01, *** *p* < 0.001. Correlation heatmaps were visualized with ggplot2 (v3.5.1; CRAN, R Foundation for Statistical Computing, Vienna, Austria).

### 4.20. Drug Sensitivity Prediction and Molecular Docking of Core Targets

Drug sensitivity was predicted using the calcPhenotype function of the R package oncoPredict (v1.2; Huang Laboratory, University of Minnesota, Minneapolis, MN, USA) based on bulk RNA-seq data. Predictions were generated using ridge regression models trained on pharmacogenomic datasets integrating gene expression and drug response data from the GDSC2 and CTRP v2 databases, as curated by the oncoPredict development team (OSF; Center for Open Science, Charlottesville, VA, USA; https://osf.io/c6tfx/, accessed on 12 December 2025).

Structural data of candidate compounds and core target proteins were obtained from the PubChem and Protein Data Bank (RCSB PDB; Rutgers University, Piscataway, NJ, USA) databases, respectively (https://www.rcsb.org/, accessed on 25 December 2025). Molecular docking was performed using CB-Dock2 (http://183.56.231.194:8001/cb-dock2/index.php, Sichuan University, Chengdu, China), which automatically preprocessed ligands and protein structures, including hydrogen addition, removal of water molecules, and energy minimization. Docking was carried out using AutoDock (v1.2.5, The Scripps Research Institute, La Jolla, CA, USA) Vina, and the binding pose with the lowest Vina score was selected as the representative conformation [94]. Three-dimensional and two-dimensional representations of the ligand–receptor complexes were visualized using Discovery Studio 2025.

### 4.21. Statistical Analysis

Statistical analysis was performed using R (v.4.4.1; R Foundation for Statistical Computing, Vienna, Austria). Comparisons between groups were conducted using χ^2^ tests or Fisher’s exact test for categorical variables. Student’s *t*-tests, Wilcoxon rank-sum tests, and ANOVA were used for continuous variables. Paired *t*-tests were used for paired comparisons. Survival analyses were conducted using log-rank tests. *p*  <  0.05 was considered to be statistically significant. Asterisk symbols indicate levels of significance as follows: * *p* < 0.05, ** *p* < 0.01, *** *p* < 0.001, **** *p* < 0.0001.

## 5. Conclusions

In conclusion, this study systematically delineates the mutation specific molecular features of TIME, identifies CM2 and CM5 as key invasive subtypes, and proposes the DMSP.sig as a quantitative tool for prognostic prediction and individualized therapy. These findings deepen our understanding of immune evolution in driver-mutant NSCLC and provide a solid rationale for the development of precision immunotherapeutic strategies.

## Data Availability

All data used in this study are publicly available from GEO and TCGA. The datasets analyzed are existing publicly accessible datasets, and detailed information is provided in Table S1. The scRNA-seq datasets used in this study include GSE171145, GSE131907, GSE148071, GSE202371, and GSE136246. In addition, we included scRNA-seq data from the study with PMID: 35027529, as well as data from the Code Ocean capsule (CO.0121060.V1). Spatial transcriptomics data were obtained from the publicly available EMBL-EBI database, including dataset E-MTAB-13530.

## Supplementary Materials

The following supporting information can be downloaded at: https://www.mdpi.com/article/10.3390/ijms27093997/s1.

## Code Availability Statement

The R scripts used for data processing, analysis, and visualization in this study are publicly available at: https://github.com/Zhangyunpeng1987/NSCLC-DMSPsig (accessed on 24 April 2026). The repository includes scripts corresponding to all figures presented in the manuscript and is organized by figure to facilitate reproducibility. A session information file is also provided to ensure transparency of the computational environment.

## Abbreviations

The following abbreviations are used in this manuscript:

NSCLC	Non-small cell lung cancer
TIME	Tumor immune microenvironment
CM	Cellular module
DMSP.sig	Driver mutation-specific prognostic signature
TF	Transcriptional regulator
NGS	Next-generation sequencing
EGFR	Epidermal growth factor receptor
ALK	Anaplastic lymphoma kinase
ScRNA-seq	Single-cell RNA sequencing
TAM	Tumor-associated macrophage
EGFR-BM	EGFR brain metastases
EMT	Epithelial–mesenchymal transition
UPR	Unfolded protein response
GE	Gene element
TLS	Tertiary lymphoid structure
CNV	Copy number variation
HR	Hazard ratio
RS	Risk Score
DC	Dendritic cell
ICI	Immune checkpoint inhibitor
PCA	Principal component analysis
DEGs	Differentially expressed genes
GO	Gene Ontology
BP	Biological process
ANOVA	Analysis of variance
FDR	False discovery rate
PDB	Protein Data Bank
GGN	Ground-glass nodule
PSN	Part-solid nodule
APC	Antigen-presenting cell
