# Supplementary material for: Coordinated Multicellular Immune Programs and Drug Targets Revealed by Single-Cell Analysis in Driver-Mutated NSCLC
Source: Int J Mol Sci. 2026 Apr 29;27(9):3997. doi: 10.3390/ijms27093997 (PMC13163347; doi:10.3390/ijms27093997)
Supplement: Supplementary file 1 [file ijms-27-03997-s001.zip › Supplementary File.pdf]

Supplementary Figures

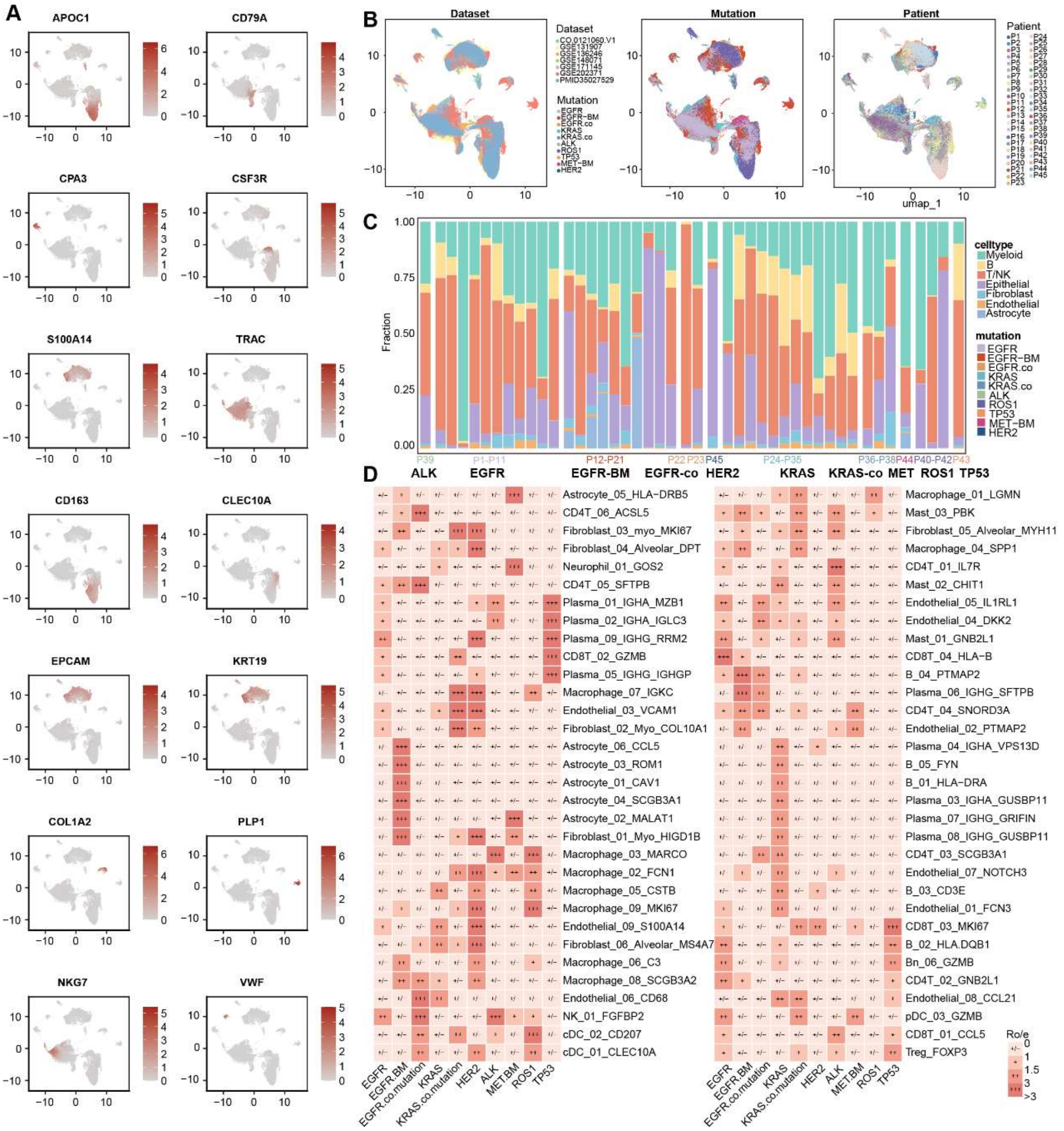

Fig.S1. Cellular landscape and functional features of driver-mutated NSCLC across patients and datasets

A) Density UMAP plots of canonical marker genes across cell populations. B) UMAPs by dataset, driver mutation, and patient. C) Stacked bar plots of major cell types per patient. D) Heatmap of 64 TIME cluster distributions across driver-mutated NSCLC samples.

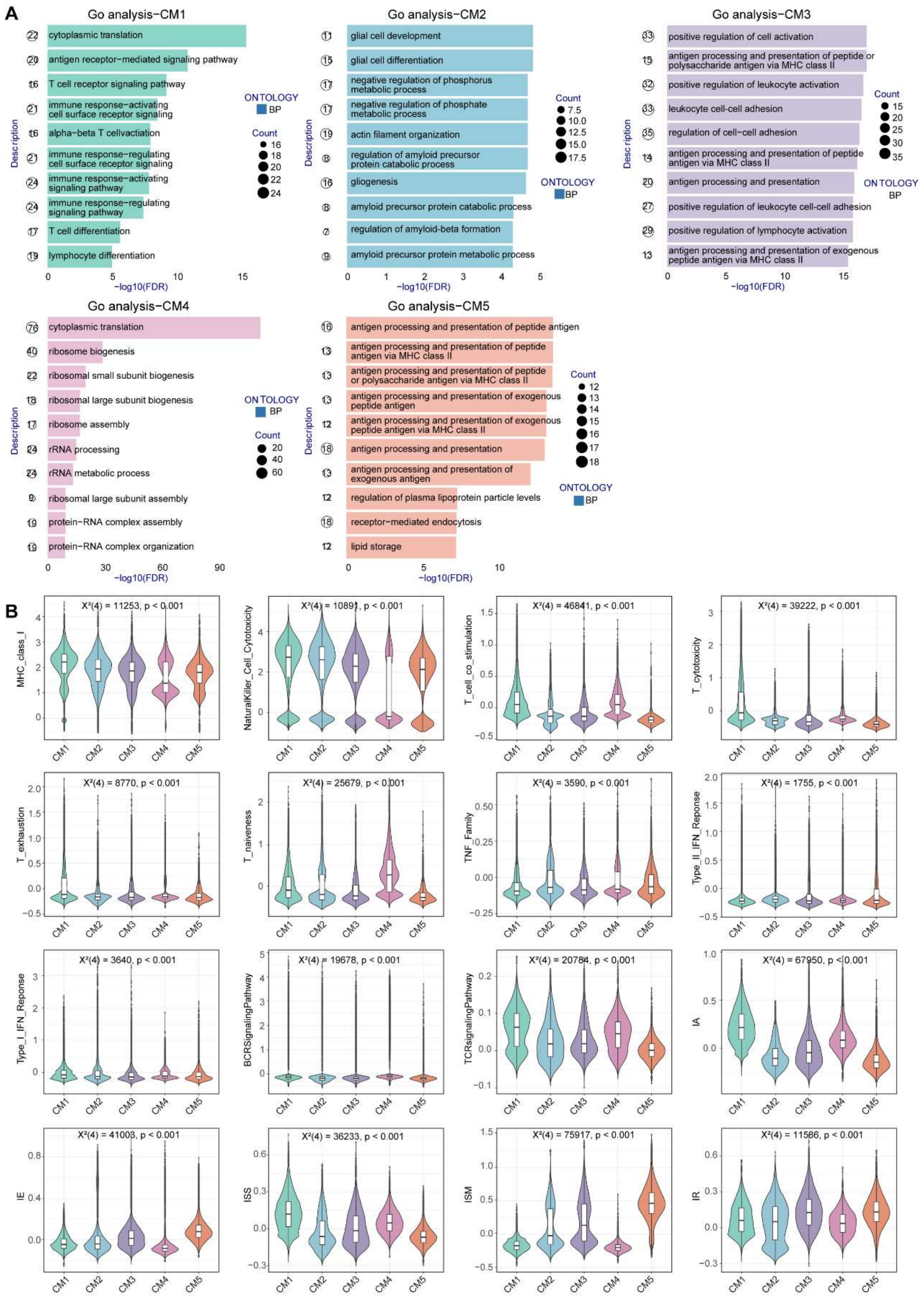

Fig.S2. Major functions and immune characteristics of TIME subtypes

A) Top 10 most enriched Gene Ontology Biological Process (GO-BP) terms for each of the five TIME subtypes. **B)** Violin plots showing the scores of other immune gene sets across TIME subtypes. Chi-square test for statistical comparison.

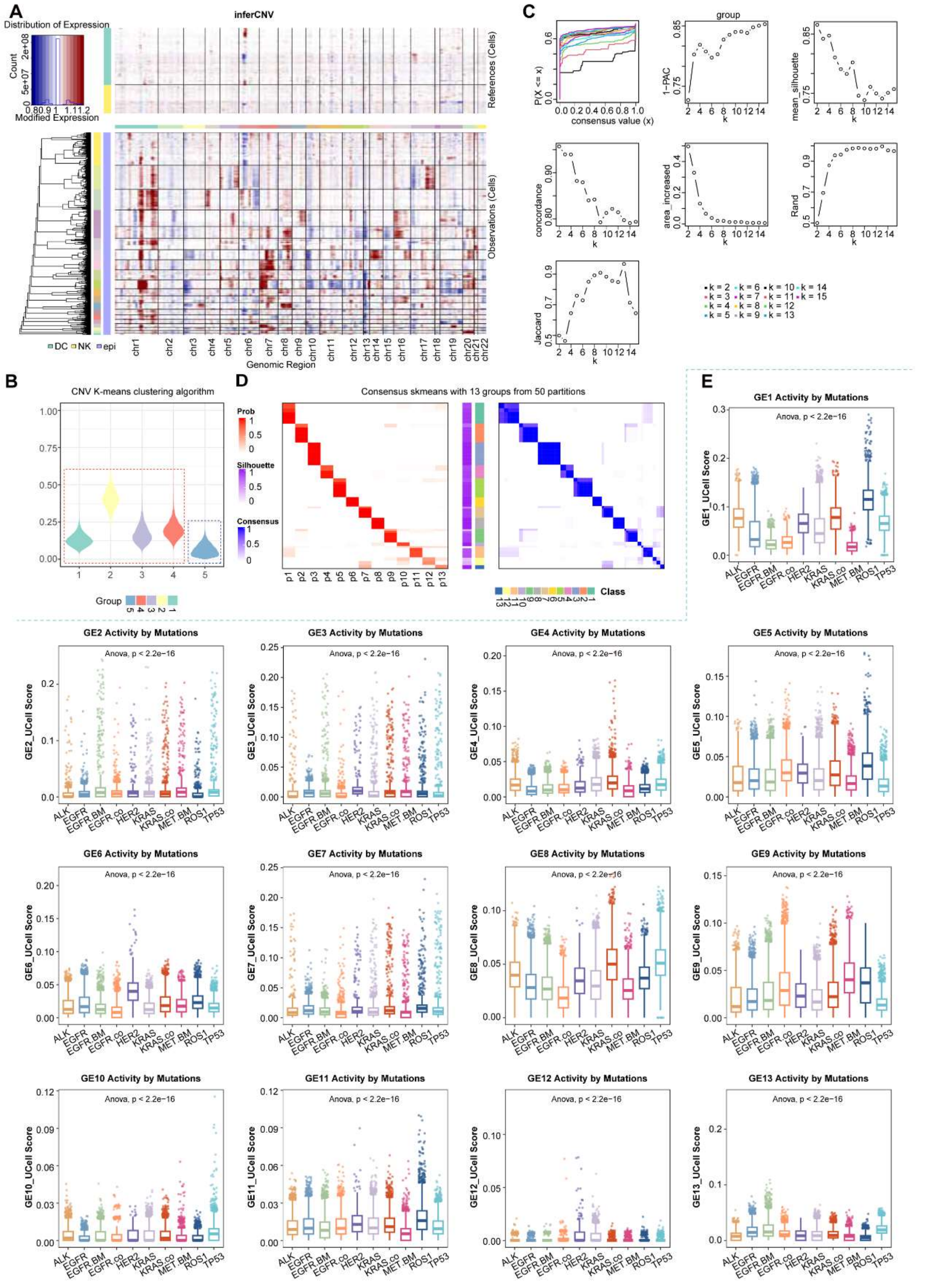

Fig.S3. Identification of TIME-associated malignant features

A) Heatmap showing CNV levels of epithelial cells inferred by inferCNV, with DC and NK cells used as reference. **B)** Violin plot illustrating CNV-based clustering using the K-means algorithm; red boxes indicate inferred malignant cells, and blue boxes denote normal cell populations. **C)** Evaluation of optimal cluster number for cancer cell GEs using multiple indices.

D) Heatmap showing the results of consensus clustering, displaying the probability (p1–p13) of each generated signature being assigned to one of the 13 classes. Silhouette scores are shown for each class or GE. **E)** Boxplots showing the distribution of representative GE signature scores across NSCLC types with different driver mutations. Statistical significance was assessed using ANOVA.

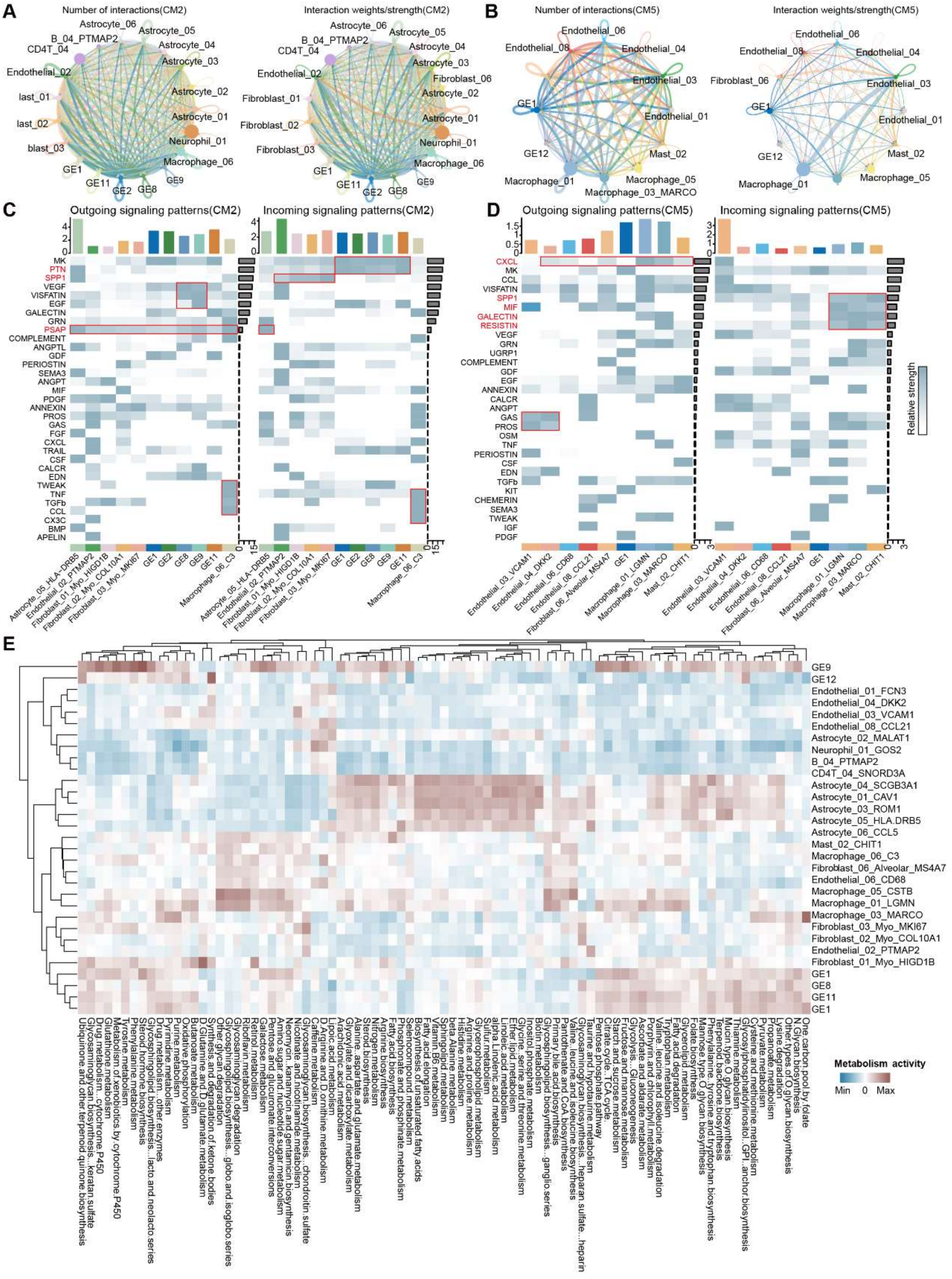

Fig.S4. Overview of cell–cell communication and metabolic patterns

A) An overview of cell–cell communication in CM2 and their associated GEs clusters. Circle size is proportional to the number of cells in each cell group. Edge thickness indicates the number (left) and the strength (right) of interaction between populations. The loops indicate cell types. **B)** An overview of cell−cell communication in CM5 and their associated GEs clusters. **C)** Heatmaps showing signaling pathways involved in the cell-cell communication processes between CM2 and their associated GEs clusters. **D)** Heatmaps showing signaling pathways involved in the cell-cell communication processes between CM5 and their associated GEs clusters. **E)** Heatmap showing metabolic pathway activity across distinct cell populations within CM2, CM5, and their associated GEs clusters.

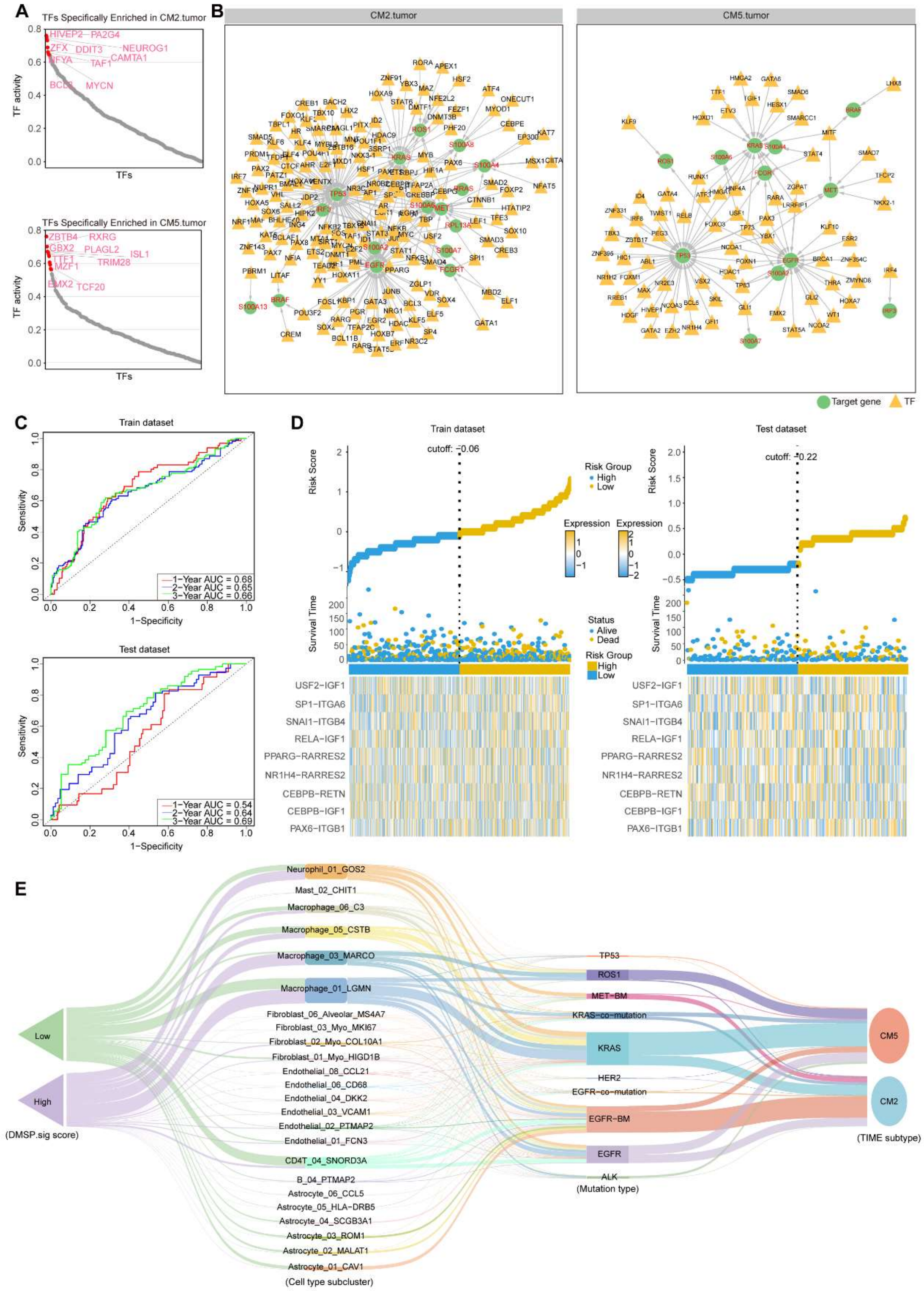

Fig.S5. The core regulons of GEs from tumor cells in both CM2 and CM5

A) Scatter plots showing highly active TFs in CM2-related cancer cells (top) and CM5-related cancer cells (bottom). The top 10 TFs are highlighted in red. **B)** Malignant regulatory networks within CM2-related cancer cells (left) and CM5-related cancer cells (right). Circular nodes indicate HR-related ligand/receptor genes, and triangular nodes denote TIME-specific highly

active TFs. **C)** ROC curves illustrating the predictive performance of the risk score for 1-, 3-, and 5-year overall survival in the training set (top) and validation set (bottom). **D)** Feature distribution of model-derived risk scores in the training cohort (left) and validation cohort (right). From top to bottom: scatter plot of risk scores, scatter plot of survival status, and clustering plot of patients. **E)** Sankey diagram illustrating the associations of the model-derived risk score and its nine constituent regulators with TIME cell subpopulations and driver mutation subtypes of NSCLC. The width of each flow represents the strength of the association.

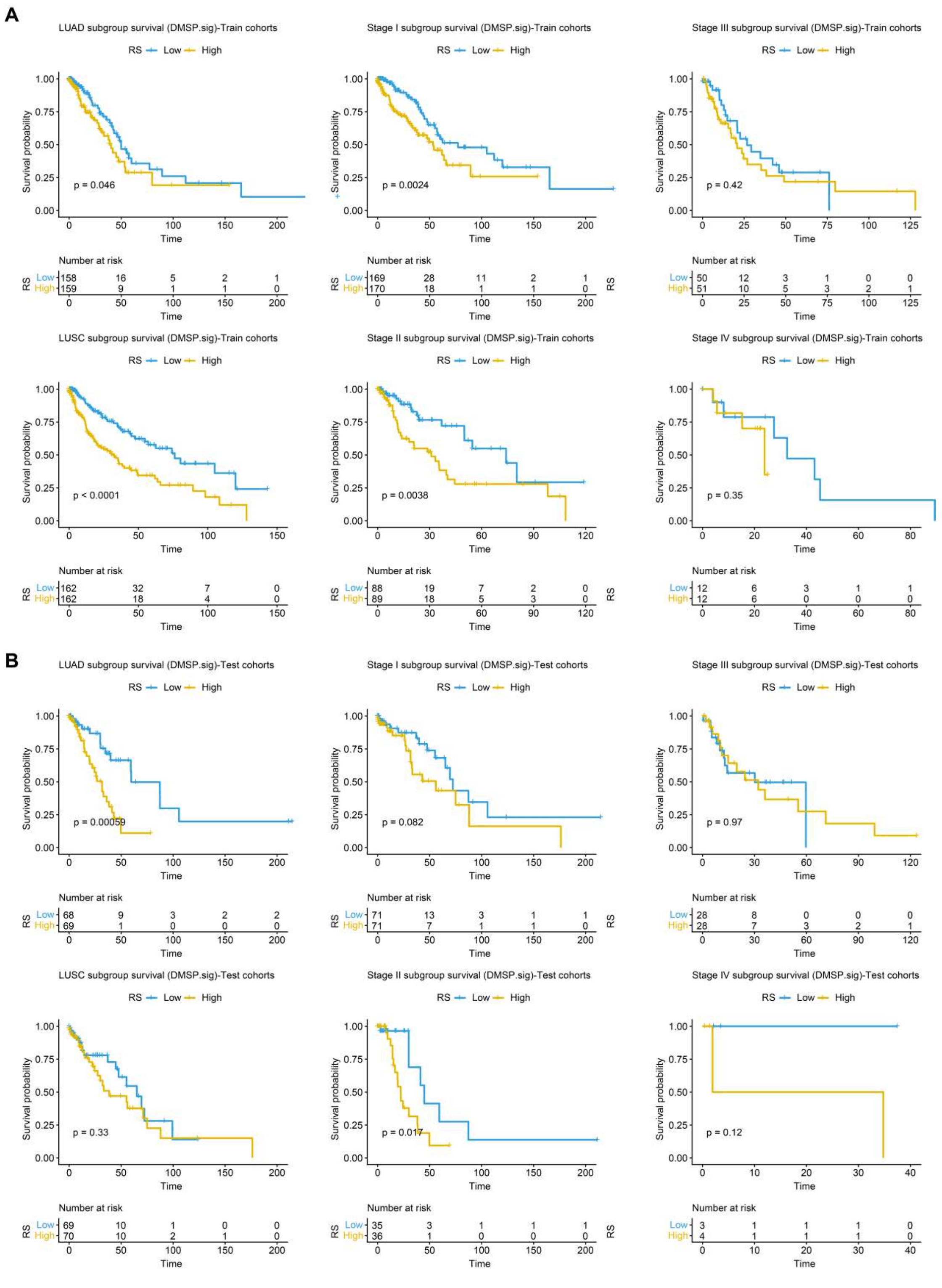

Fig. S6. Prognostic performance of the DMSP.sig model across clinical subgroups in the training and test cohorts

A) Kaplan–Meier survival analysis of patients in the training cohort stratified by clinical subgroups, including LUAD, LUSC, and tumor stages (Stage I–IV). Patients in each subgroup were divided into high- and low-risk groups based on the median DMSP.sig score. The number of patients in each subgroup is indicated in the risk tables. **B)** Kaplan–Meier survival analysis of patients in the test cohort using the same stratification strategy as in the training cohort.

Patients were grouped by histological subtype (LUAD, LUSC) and tumor stage (Stage I–IV), and classified into high- and low-risk groups based on the median DMSP.sig score. The number of patients in each subgroup is shown in the corresponding risk tables.

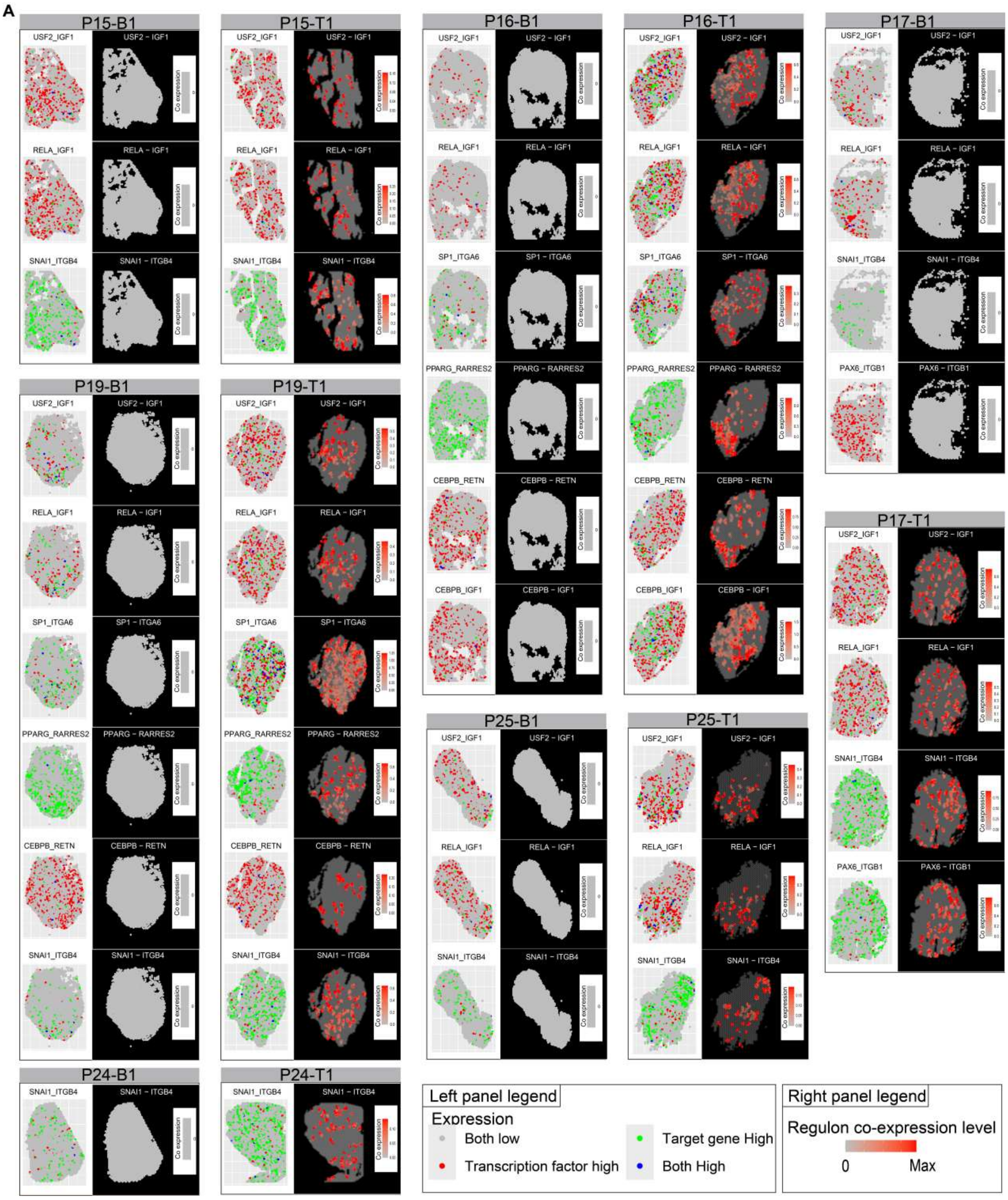

Fig. S7. Spatial transcriptomic validation of prognostic regulons using independent NSCLC cohorts

A) Spatial expression patterns of prognostic regulons (transcription factor–target gene pairs) in an independent spatial transcriptomics dataset (E-MTAB-13530) obtained from the EMBL-EBI database. Six paired samples from NSCLC patients were included, consisting of matched adjacent normal tissues (P15-B1, P16-B1, P17-B1, P19-B1, P24-B1, P25-B1) and tumor tissues (P15-T1, P16-T1, P17-T1, P19-T1, P24-T1, P25-T1). For each sample, the left panel shows the spatial expression of transcription factors (red) and target genes (green), as well as spots with no expression (gray) or co-expression (blue). The right panel displays regulon-level co-localization signals, where higher intensity (red) indicates stronger co-expression of transcription factors and target genes within the same spatial spots.
